# Transcriptomic Profiling of Soybean in Response to High-Intensity UV-B Irradiation Reveals Stress Defense Signaling

**DOI:** 10.3389/fpls.2016.01917

**Published:** 2016-12-19

**Authors:** Min Young Yoon, Moon Young Kim, Sangrae Shim, Kyung Do Kim, Jungmin Ha, Jin Hee Shin, Sungtaeg Kang, Suk-Ha Lee

**Affiliations:** ^1^Department of Plant Science and Research Institute of Agriculture and Life Sciences, Seoul National UniversitySeoul, South Korea; ^2^Plant Genomics and Breeding Institute, Seoul National UniversitySeoul, South Korea; ^3^Center for Applied Genetic Technologies, University of GeorgiaAthens, GA, USA; ^4^Department of Crop Science and Biotechnology, Dankook UniversityCheonan, South Korea

**Keywords:** UV-B stress resistance, phosphatidic acid, diacylglycerol kinase, TIR-NBS-LRR, transcriptomic profiling, soybean

## Abstract

The depletion of the ozone layer in the stratosphere has led to a dramatic spike in ultraviolet B (UV-B) intensity and increased UV-B light levels. The direct absorption of high-intensity UV-B induces complex abiotic stresses in plants, including excessive light exposure, heat, and dehydration. However, UV-B stress signaling mechanisms in plants including soybean (*Glycine max* [L.]) remain poorly understood. Here, we surveyed the overall transcriptional responses of two soybean genotypes, UV-B-sensitive Cheongja 3 and UV-B-resistant Buseok, to continuous UV-B irradiation for 0 (control), 0.5, and 6 h using RNA-seq analysis. Homology analysis using UV-B-related genes from *Arabidopsis thaliana* revealed differentially expressed genes (DEGs) likely involved in UV-B stress responses. Functional classification of the DEGs showed that the categories of immune response, stress defense signaling, and reactive oxygen species (ROS) metabolism were over-represented. UV-B-resistant Buseok utilized phosphatidic acid-dependent signaling pathways (based on subsequent reactions of phospholipase C and diacylglycerol kinase) rather than phospholipase D in response to UV-B exposure at high fluence rates, and genes involved in its downstream pathways, such as ABA signaling, mitogen-activated protein kinase cascades, and ROS overproduction, were upregulated in this genotype. In addition, the DEGs for TIR-NBS-LRR and heat shock proteins are positively activated. These results suggest that defense mechanisms against UV-B stress at high fluence rates are separate from the photomorphogenic responses utilized by plants to adapt to low-level UV light. Our study provides valuable information for deep understanding of UV-B stress defense mechanisms and for the development of resistant soybean genotypes that survive under high-intensity UV-B stress.

## Introduction

The depletion of the ozone layer in the stratosphere has led to increased levels terrestrial ultraviolet B (UV-B, 280–315 nm) radiation until now since the late 1970s (Searles et al., 2001; Austin and Wilson, 2006). Furthermore, it is causing periodic or unpredictable spikes in UV-B intensity in the polar and temperate zones (Kerr and McElroy, 1993). The Montreal Protocol has been effective in stabilizing UV-B exposure since the mid-1990s but the recovery of the ozone layer to pre-1980 levels may require several decades (McKenzie et al., 2011). UV-B photons can function as both environmental stressors and developmental signals in plants (Britt, 1996). Since sunlight is required for photosynthesis, increased UV-B intensity inevitably threatens plant viability, as most vascular plants cannot adapt to such an influx of UV-B (Jordan, 2002; Paul and Gwynn-Jones, 2003). High level UV-B radiation causes physiological damage, such as reduced photosynthetic capacity and impaired pollen fertility, as well as morphological changes including plant stunting, leaf discoloration, and reduced biomass and seed yields (Frohnmeyer and Staiger, 2003; Lytvyn et al., 2010).

Despite the complex effects of UV-B radiation on plants, recent studies have revealed a series of components implicated in UV-B-specific photomorphogenic (non-damaging) regulation in the circadian rhythm pathway (Ulm and Nagy, 2005). These components include the UV-B photoreceptor UV RESISTANCE LOCUS8 (UVR8), the E3 ubiquitin ligase (transducin/WD40 repeat-like superfamily protein) CONSTITUTIVELY PHOTOMORPHOGENIC1 (COP1), the basic leucine-zipper transcription factor ELONGATED HYPOCOTYL5 (HY5), and its interacting partner LONG HYPOCOTYL5-LIKE (HYH) (Ulm, 2003). In addition, *CHALCONE SYNTHASE* (*CHS*) and other genes involved in the biosynthesis of secondary metabolites (e.g., flavonoids and phenylpropanoids) are positively activated in response to UV-B radiation (Heijde and Ulm, 2012). On the other hand, UV-B radiation above ambient level, like other environmental stresses, elicits nonspecific (genotoxic) damage responses that trigger stress defense signaling (Jenkins, 2009). In *Arabidopsis thaliana*, reactive oxygen species (ROS) production are induced by high dose UV-B irradiation (Heijde and Ulm, 2013; Parihar et al., 2015). Genes activated by stress responses to high level UV-B are distinct from those playing a role in photomorphogenesis specific to low dose UV-B (Müller-Xing et al., 2014). However, the mechanism by which UV-B stress triggers the intracellular defense signaling pathway remains poorly understood (Müller-Xing et al., 2014).

Annual soybean [*Glycine max* (L.) Merr.] is one of the most important crops that were used as food, energy, and industrial resources worldwide. Only a few studies have compared the morphological, anatomical, and biochemical differences between UV-B-sensitive and -resistant soybean cultivars (Essex vs. Williams) in response to enhanced UV-B radiation (Murali et al., 1988; Jordan, 1993, 2002; Grant et al., 2010). Recently, quantitative trait loci associated with resistance to supplementary UV-B treatment were localized between Satt495 and Satt238 on chromosome 19 using a recombinant inbred line population of Keunol (UV-B sensitive) x Iksan10 (UV-B resistant) (Shim et al., 2015; Lee et al., 2016). Meanwhile, our previous study showed the evaluation of a total of 140 genotypes including 94 *G. max* and 46 *Glycine soja* accessions for their sensitivity to supplemental UV-B radiation, resulting in the selection of the most resistant Buseok and the most sensitive Cheongja 3 (Kim et al., 2015). This UV-B resistant genotype Buseok will be a valuable plant material not only for breeding of UV-B resistant cultivars but for molecular genetic study on UV-B resistance mechanisms.

The present study was conducted to figure out how soybean copes with UV-B of high intensity as environmental stress at a transcriptomic level and to verify if UV-B defense pathways are dependent on the UV-B-specific photomorphogenic regulation or not. We surveyed the overall transcriptional responses of two soybean genotypes, UV-B-sensitive Cheongja 3 and UV-B-resistant Buseok, to continuous UV-B irradiation for 0 (control), 0.5, and 6 h at high fluence rates. We compared the transcript abundance between the se genotypes in response to supplementary UV-B irradiation, as well as between control and UV-B treatments in each genotype. The differentially expressed genes (DEGs) were subjected to further filtering using a set of UV-B related genes from *A. thaliana* to identify DEGs that are most likely involved in UV-B stress defense. Furthermore, the biological functions of four DEGs were confirmed using *A. thaliana* knock-out mutants. The results of this study provide insights into the molecular basis of the capacity for plants to tolerate UV-B stress at high fluence rates.

## Materials and methods

### Plant materials and growth conditions

UV-B-sensitive Cheongja 3 and UV-B-resistant Buseok (IT162669), which were identified as the most sensitive and resistant soybean genotypes to supplementary UV-B irradiation, respectively (Kim et al., 2015), were used in this study. Under 6 h of UV-B treatment, Buseok had only one or two slightly yellowish leaves and still showed vigorous growth, whereas in Cheongja 3, most leaves turned yellow with red spots, ultimately leading to defoliation (Figure S1). Seeds of both soybean genotypes were planted in plastic pots (18 cm diameter, 20 cm deep) in a 1:1:1 mixture of field soil, desalinated sand, and commercial peat soil in a greenhouse at Seoul National University Experimental Farm. One plant per pot was grown under a natural photoperiod of 11.5–14.5 h per day using standard cultivation methods.

### UV-B treatment

For UV-B treatment, soybean plants were divided into three groups; no UV-B treatment (0 h) as a control, 0.5- and 6-h UV-B treatments). Supplementary UV-B irradiation was conducted at V4 growth stage as previously described (Kim et al., 2015). UV-B irradiation began at 9:00 am, and the plants were exposed to prolonged UV-B stress for 0.5 and 6 h. The intensity of 0.5 h UV-B irradiation was equivalent to 11.5 kJ/m^2^ daily soybean UV-B biological effective dose (UV-B_BE_), and the 6 h treatment was equivalent to a dose of irradiation that was 12-times higher than daily UV-B_BE_ (Caldwell, 1971). For Illumina RNA sequencing, the uppermost trifoliate leaves were collected from UV-B-treated and non-treated plants. At each sampling time, the leaf tissues from four pots per group of each genotype were pooled together as one biological replicate, frozen immediately in liquid nitrogen, and stored at −80 until use. Thus, a total of six leaf samples (three treatments [0, 0.5, and 6 h] × two genotypes [Cheongja 3 and Buseok] × one biological replicate) were collected for RNA-seq analysis.

### RNA isolation and RNA sequencing using illumina HiSeq

Total RNA was extracted from the leaf samples using TRIzol reagent (Invitrogen, Carlsbad, CA, USA) according to the manufacturer's instructions. The cDNA library for Illumina HiSeq 2000 sequencing was constructed using an mRNA-seq sample preparation kit (TruSeq® RNA Sample Prep Kit v2, Cat.8207217, Illumina Inc., San Diego, CA, USA), including mRNA purification, cDNA synthesis, end-repair of cDNA, adaptor ligation, and cDNA amplification. Six cDNA libraries were pooled at equal molar concentration for loading onto two lanes of an Illumina flow cell and then merged for data analysis. Sequencing runs were performed in paired-end mode for 10l cycles using the Illumina HiSeq 2000 platform.

### Read alignment and RNA-Seq analysis

The 100 bp paired-end sequence reads were mapped to the soybean reference genome (Glyma v1.1) from Phytozome (http://www.phytozome.net/soybean) using Bowtie (http://bowtie-bio.sourceforge.net/index.shtml) and TopHat (http://ccb.jhu.edu/software/tophat/index.shtml) in default modes (Langmead et al., 2009; Trapnell et al., 2009). The Cufflinks program was used to assemble gene transcripts and to normalize transcript abundance in terms of fragments per kilobase pair of transcript per million mapped reads (FPKM) (Trapnell et al., 2012). The Cuffdiff program (http://cufflinks.cbcb.umd.edu/index.html) was used to test statistically significant differences in transcript expression in seven pairs of comparisons, which consisted of three comparisons of Cheongja 3 vs. Buseok under control conditions and two UV-B treatments, and four comparisons of control vs. UV-B treatments in the two genotypes (Figure S2). Significant DEGs were detected using the following criteria: (i) absolute fold-change > 1 and (ii) *q*-value (false discovery rate [FDR]) <0.05 and *p*-value < 0.004. R studio (https://www.rstudio.com/, version 3. 3. 0) was used to run custom R scripts to perform hierarchical cluster analysis of DEG expression and to construct heat maps.

### Survey of *A. thaliana* UV-B related genes

UV-B related genes from *A. thaliana* were surveyed using Gene Ontology (GO) (http://amigo.geneontology.org/amigo/search/ontology?q=UV-B) and The Arabidopsis Information Resource (TAIR) (https://www.Arabidopsis.org/) database. In addition, a list of *A. thaliana* genes shown to be involved in the UV-B response was compiled from the literature; 194 UV-B-related *A. thaliana* genes were ultimately obtained (Figure S2, Supplementary Table 1). To detect genes that function in networks with the UV-B related genes, the set of selected UV-B related genes was analyzed with Aranet (http://www.functionalnet.org/aranet/), a probabilistic functional gene network for *A*. *thaliana*, resulting in the identification of 4666 genes predicted to respond directly or indirectly to UV-B (Figure S2, Supplementary Table 2). A total of 15,074 soybean homologs of these *A. thaliana* genes were identified using Blast analysis. Based on this soybean gene list, DEGs putatively involved in the defense response to UV-B stress were identified (Figure S2).

### Functional classification of DEGs by BINGO and KEGG

To better understand the biological functions of the DEGs in response to UV-B, enrichment of GO categories among the DEGs was assessed using BINGO software (http://www.psb.ugent.be/cbd/papers/BiNGO/Home.html) (Maere et al., 2005). Significantly over-represented GO categories were visualized in Cytoscape (http://www.cytoscape.org). The biochemical pathways involving the DEGs were predicted using Kyoto Encyclopedia of Genes and Genomes (KEGG) (http://www.genome.jp/kegg/).

### Quantitative reverse-transcription PCR validation of DEGs

Gene-specific primers were designed based on the nucleotide sequences of the chosen DEGs for qRT-PCR analysis using Primer3 software (http://primer3plus.com/web_3.0.0/primer3web_input.htm) (Supplementary Table 3). Total RNA from six samples (Cheongja 3 and Buseok; control, 0.5, and 6 h UV-B treatments) was used to synthesize cDNA using a Bio-Rad iScript™ cDNA Synthesis Kit (Cat. 170-8891, Hercules, CA, USA). The synthesized cDNAs were used for qRT-PCR with a Bio-Rad iQ™ SYBR Green Supermix Kit (Cat. 170-8882) using a LightCycler® 480 (Roche Diagnostics, Laval, QC, Canada). The qRT-PCR reaction mixtures (total volume of 20 μl) contained 100 ng of cDNA, each primer at 300 μM, 8 μl of sterile water, and 10 μl of Bio-Rad iQ™ SYBR Green Supermix. The amplification conditions were as follow: 5 min denaturation at 95°C followed by 40 cycles of 95°C for 10 s and 60°C for 1 min. The samples were analyzed in triplicate to ensure statistical significance, and the tubulin gene was used as a reference gene for normalization of target gene expression in soybean. Data were analyzed based on the stable expression level of the reference gene according to the method of Livak and Schmittgen (2001). To assess treatment effects on soybean genotypes, significances were analyzed with Fisher's least significant difference tests (*P* < 0.05) using the Statistical Analysis System (SAS 9.4 for window, SAS Institute Inc., Cary, NC, USA).

### Functional validation of DEGs using arabidopsis knock-out mutants

Seeds of knock-out mutants for the target genes were obtained from the Arabidopsis Biological Resource Center (ABRC, http://abrc.osu.edu/) at Ohio State University, USA. Seven mutant lines were utilized, including mutants for five members of TIR-NBS-LRR family (AT1G64070.1; SALK_042846C, AT5G17680.1; SALK_004241C, AT5G36930.1; SALK_124056C, AT5G41540.1; SALK_034471C, and AT4G36150.1; SALK_084909C), one DGK mutant (AT5G07920.1; SALK_033664C), and one PIP5K mutant (AT1G34260.1; SALK_047604C). To identify homozygous mutants for the target genes, seeds incubated at 4°C were sown in commercial peat soil and grown under 16/8 h light conditions in a growth chamber for 14 days. Homozygous mutant plants were confirmed by RT-PCR using primers designed based on the inserted T-DNA and target gene DNA sequences, resulting in the identification of only four knock-out mutants (Figure S3; Supplementary Table 4). Both knock-out mutant and Columbia ecotype (Col-0) seeds were sterilized and incubated at 4°C in the dark for 4 days to synchronize germination and to ensure uniform growth, followed by cultivation under 16 h white fluorescent light for 7 days in a growth chamber. As a pilot UV-B irradiation test to determine suitable irradiation times for *A. thaliana*, wild-type (Col-0) plants were subjected to different UV-B treatments (1, 2, 4, and 8 h); 4 h UV-B treatment was determined to be suitable (Figure S4). Seven-days-old knock-out mutants were treated with 4 h UV-B irradiation at 22°C. For qRT-PCR analysis, rosette leaves were collected from UV-B-treated and non-treated mutant plants, frozen immediately in liquid nitrogen, and stored at −80°C until use. The specific primers for qRT-PCR were designed using primer 3 (Supplementary Table 5).

## Results

### RNA-seq analysis and DEG identification related to UV-B stress in Cheongja 3 and Buseok

To compare transcriptomic variation in the soybean lines in response to UV-B stress, Cheongja3 and Buseok leaves were collected after continuous UV-B exposure at a high fluence rate for 0 (control), 0.5, and 6 h, with a total of six samples. Using Illumina HiSeq 2000 system, 0.3 billion 100 bp paired-end reads were produced, ranging from 47 to 62 million reads per sample (Supplementary Table 6), which were mapped against the soybean reference sequence. The RNA-seq analysis workflow depicted in Figure S2 was implemented to analyze the sequencing data. Of the total reads, 78% were properly mapped to the reference sequence, resulting in approximately 25-fold average coverage (Supplementary Table 6). About 20% unmapped reads appears to be attributable to either misassembled or absent sequences in the reference assembly.

To identify DEGs related to UV-B stress, we compared transcript abundances among the six samples from two soybean genotypes differing in UV-B tolerance based on FPKM values. Two-way comparison data analysis was used to investigate transcriptomic variations, representing the comparison (i) between Cheongja 3 and Buseok by UV-B treatment and (ii) between control and UV-B treatments by genotype (Figure S2). To further identify DEGs in response to supplementary UV-B irradiation, we then utilized a set of 4666 *A. thaliana* genes predicted to be implicated in UV-B stress defense, either directly or indirectly, via Aranet (Figure S2; Supplementary Tables 1, 2). Homology comparative analysis revealed that 1875 DEGs detected in the Cheongja 3 vs. Buseok comparison were orthologs of UV-B-related Arabidopsis genes (Figure S2). In the control vs. treatment comparison, 228 DEGs in Buseok and 129 in Cheongja 3 were found to be homologous to the UV-B related Arabidopsis genes (Figure S2; Supplementary Table 7–9).

**Figure 1:**
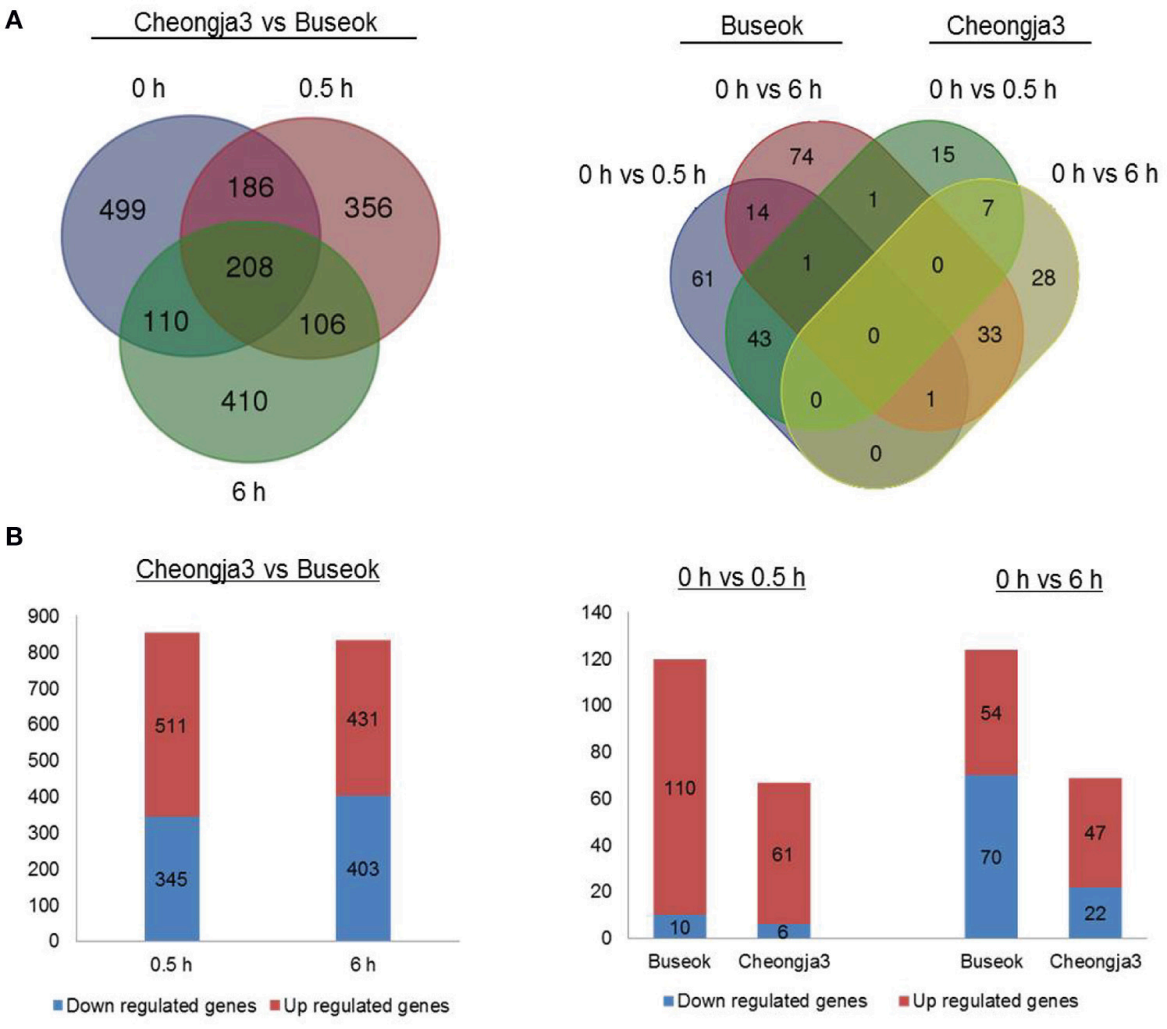
**Number of differentially expressed genes (DEGs). (A)** Left: DEGs between Cheongja 3 and Buseok under control, 0.5, and 6 h UV-B treatments. Right: DEGs between the control and UV-B treatments in Cheongja 3 and Buseok. **(B)** Up- and down-regulated DEGs detected in each comparison.

Using the final sets of DEGs, we generated Venn diagrams to identify treatment- or genotype-specific DEGs and common DEGs (Figure 1A). A larger number of DEGs were specific to each genotype and each UV-B treatment time. In the Cheongja 3 vs. Buseok comparison, 499 DEGs specific to the controls were considered to be due to differences in the genetic backgrounds between the genotypes and were thus excluded from further functional classification. Of the 1376 DEGs in Buseok relative to Cheongja 3 (Supplementary Table 9), 511 were upregulated and 345 were downregulated under 0.5 h UV-B treatment (Figure 1B). Under 6 h UV-B treatment, the number of up- and downregulated DEGs was similar. The control vs. treatment comparisons by genotype showed that Buseok had more DEGs than Cheongja 3 both for 0 vs. 5 h and 0 vs. 6 h (Figure 1B). In Cheongja 3, the number of DEG is higher in upregulation than downregulation for 0 vs. 6 h. There were 149 and 50 Buseok- and Cheongja 3-specific DEGs, respectively (Figure 1A). Most DEGs in both genotypes were upregulated by 0.5 h UV-B treatment but more strongly downregulated by 6 h UV-B exposure (Figure 1B).

### Functional classification of DEGs by Bingo and KEGG

**Figure 2:**
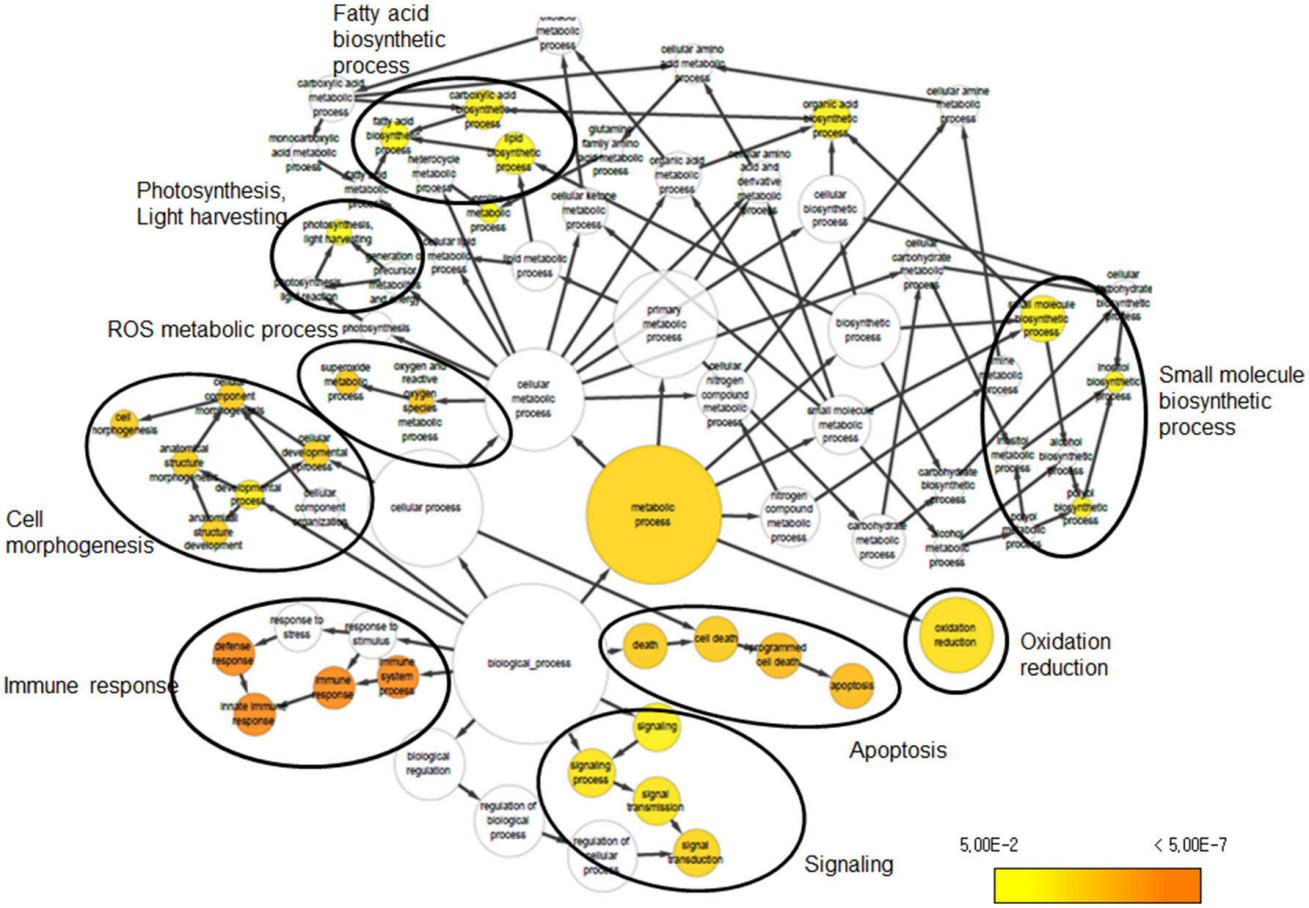
**Over-represented GO terms of DEGs that were identified from the comparison between Cheongja 3 and Buseok under UV-B treatments**.

We investigated the biological significance of the changes in transcript abundance provoked by UV-B irradiation stress in soybean. To address the functional distribution of the identified DEGs, we performed GO term enrichment using Cytoscape plug-in BINGO. GO assignment of 690 DEGs among the 1376 DEGs identified in the Cheongja 3 vs. Buseok comparison by UV-B treatment resulted in significant overrepresentation of 31 GO terms, including 17 in the biological process category and 14 in the metabolic process category (Figure 2; Supplementary Table 10). In the biological process category, over-represented GO terms were mainly classified into four clusters, as shown in Figure 2, including cell morphogenesis, immune response, signaling, and apoptosis. A large portion of the DEGs involved in the GO clusters immune response, signaling, and apoptosis were overlapping, i.e., toll/interleukin-1 nucleotide-binding-site leucine-rich repeat (TIR-NBS-LRR) genes. The GO terms over-represented in metabolic process were primarily divided into five clusters including light harvesting system, fatty acid biosynthetic process, small molecule biosynthetic process, ROS metabolic process, and oxidation reduction. From the control vs. treatment comparisons in UV-B-resistant Buseok, 176 DEGs induced by 0.5 h UV-B treatment were grouped into six GO clusters, including biological regulation, immune response, signaling, apoptosis, ion transport, and polysaccharide metabolic process (Figure S5; Supplementary Table 11). However, no GO term was over-represented by the 124 DEGs in Buseok in response to 6 h UV-B treatment. In the control vs. treatment comparisons in UV-B-sensitive Cheongja 3, 9 DEGs under 0.5 h UV-B treatment were grouped into the GO term metal ion transport (GO:0030001) and 26 DEGs under 6 h UV-B treatment were grouped into oxidation reduction (GO:0055114) (Figure S6; Supplementary Table 12). According to BINGO analysis, the GO terms involved in immune response, cell death, and signaling were the most dominant.

KEGG pathway mapping of the 1376 DEGs identified in the Cheongja 3 vs. Buseok comparison under UV-B treatments revealed the involvement of 101 biological pathways (Supplementary Table 13). The 228 and 129 DEGs in the control vs. treatment comparisons in Buseok and Cheongja 3 were assigned to 28 and 52 KEGG pathways, respectively (Supplementary Tables 14, 15). Interestingly, phosphatidylinositol signaling system (gmx04070) and mTOR signaling pathway (map04150), which are involved in environmental information processing and signal transduction, were also identified.

Based on the results of BINGO and KEGG analysis, we specifically focused on DEGs in over-represented GO and KEGG terms such as cell death and immune system, stress defense signaling, and ROS metabolism, as described below. We also investigated the expression patterns of genes in the photosystem and circadian rhythm categories.

### Photosystem and circadian rhythm

Supplemental UV-B radiation downregulates genes encoding several key photosynthetic proteins, including small subunits of Rubisco, subunits of ATP synthase, and chlorophyll a/b binding protein of the light harvesting antenna complex of photosystem II (Jenkins, 2009). In the present study, there are no photosynthetic DEGs by UV-B treatments in both Buseok and Cheongja 3 compared to the control (Supplementary Tables 7–9, 16). However, differences between the two soybean genotypes were observed in the transcript abundance of chlorophyll a/b binding protein and photosystem light harvesting complex genes under both control and elevated UV-B conditions (Supplementary Table 16). Inherent variations in the expression of two genes encoding light harvesting complex subunits were detected between Buseok and Cheongja 3 under ambient light (control). In addition, the expression patterns of six genes associated with photosystem during UV-B irradiation significantly differed between genotypes, which included two homologs (Glyma09g0826 and Glyma15g19810) for photosystem I light harvesting complex gene 6, one (Glyma04g33360) for light-harvesting chlorophyll-protein complex I subunit A4, one (Glyma16g27995) for light-harvesting chlorophyll-protein complex II subunit B1, and one (Glyma11g35130) for light harvesting complex photosystem II (Supplementary Table 16). These results indicate that the transcriptional regulation of photosynthetic genes in response to supplementary UV-B irradiation is different between soybean genotypes.

Photomorphogenic UV-B responses are specifically mediated by (but are not independent of) other aspects of light signaling, including photoreceptors and circadian rhythms. We observed significant differences in the transcript abundance of genes involved in UV-B specific photomorphogenic pathways between UV-B-resistant Buseok and UV-B-sensitive Cheongja 3. Specifically, *HY5* (Glyma08g41450) and *COP1* (Glyma14g05430) were upregulated by 6 h UV-B treatment (Supplementary Table 9). Most DEGs encoding CHS proteins, which were expressed at lower levels in Buseok compared to Cheongja 3 under control conditions, were upregulated by UV-B irradiation (Supplementary Table 16). Additional key genes involved in the circadian rhythm pathway, such as *PHYTOCHROME A, GIGANTEA PHYTOCHROME INTERACTING FACTOR 3, and PSEUDO-RESPONSE REGULATOR*, were also differentially expressed in response to supplementary UV-B exposure (Supplementary Table 16). In the control vs. treatment comparisons by genotype, *UVR8, HY5*, and *COP1* were not detected among DEGs (data not shown).

### Cell death and immune response

**Figure 3:**
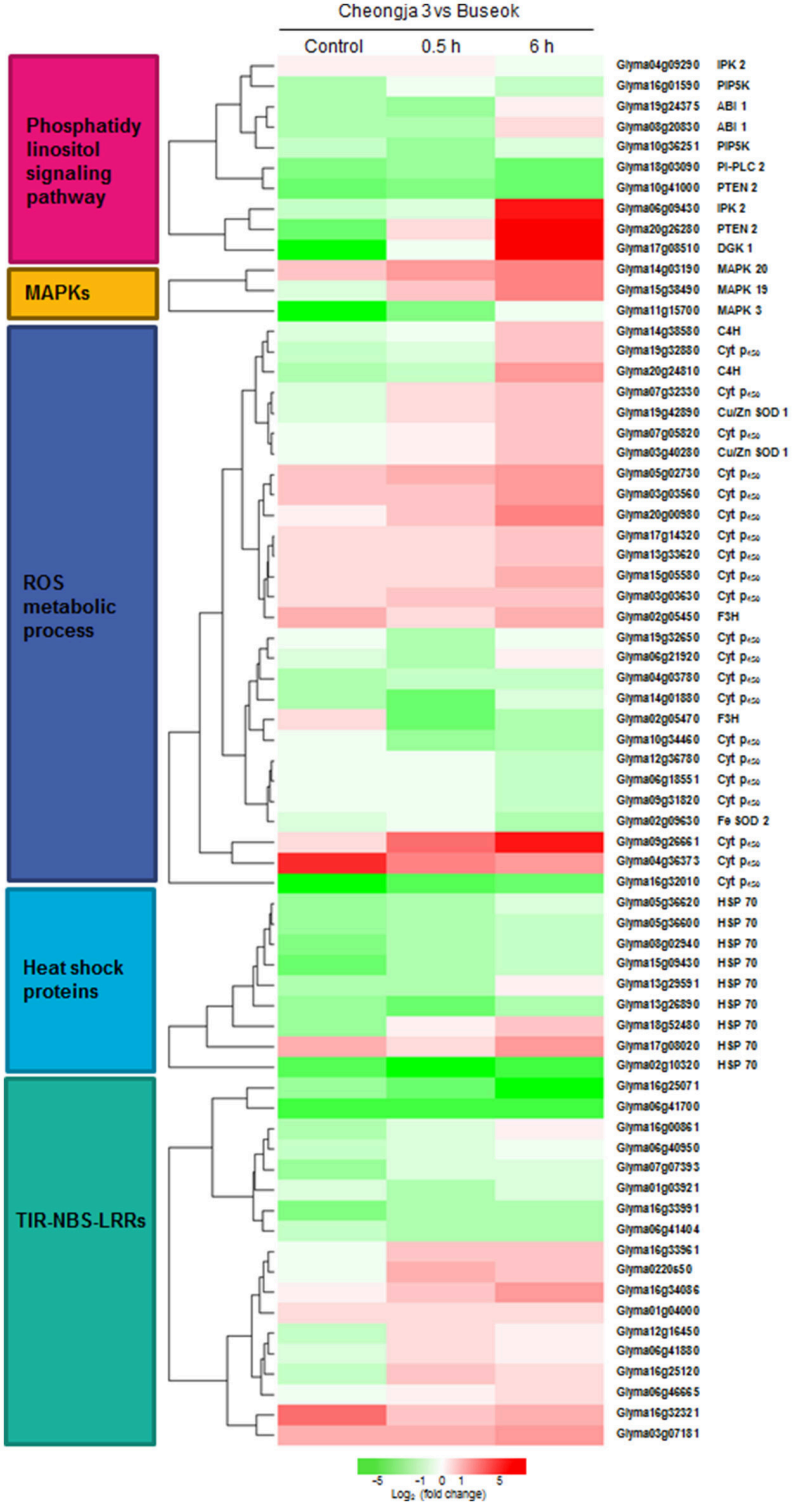
**Heatmap showing the differential expression levels of genes encoding components involved in the over-represented GO and KEGG terms between Cheongja 3 and Buseok under control, 0.5, and 6 h UV-B treatments**.

To investigate which components in defense signaling pathways are activated in response to UV-B stress, we investigated significant differences in the expression of genes participating in programmed cell death and the immune system. The comparison of Cheongja 3 vs. Buseok under UV-B treatments revealed differential expression of 20 *TIR-NBS-LRR* genes, 11 and 9 of which were upregulated in Buseok and Cheongja 3, respectively (Table 1; Figure 3). Seven additional *TIR-NBS-LRR* and two coiled-coil-nucleotide-binding-site leucine-rich repeat (*CC-NBS-LRR*) genes were induced by 0.5 h UV-B irradiation only in Buseok (Table 1), while none of these genes were induced by 6 h treatment.

**Table 1:** **Differential expressed genes encoding TIR/CC-NBS-LRRs and heat shock proteins identified by the transcriptomic comparisons of UV-B sensitive Cheongja 3 vs. resistant Buseok and control vs. UV-B treatments in Buseok**.

**Gene ID**	**Chromosome position**	**Gene definition**	**Log**_**2**_ **(fold change)**^a^			***A. thaliana*** **homolog**
**Cheongja3 vs. Buseok**			**Control**	**0.5 h**	**6 h**	
Glyma01g03921	Gm01:3390102-3396781	Disease resistance protein (TIR-NBS-LRR class), putative	−1.38	−3.62	−1.62	AT5G17680.1
Glyma01g04000	Gm01:3487914-3494204	Disease resistance protein (TIR-NBS-LRR class), putative	1.79	1.85	1.56	AT5G17680.1
Glyma0220s50	scaffold_220:14-5602	Disease resistance protein (TIR-NBS-LRR class) family	−0.56	3.09	2.29	AT5G36930.2
Glyma03g06285	Gm03:6480791-6481760	Disease resistance protein (TIR-NBS-LRR class) family	−	−	−5.18	AT5G44510.1
Glyma03g07181	Gm03:7546779-7575160	Disease resistance protein (TIR-NBS-LRR class) family	3.19	3.53	3.96	AT5G36930.2
Glyma06g40950	Gm06:44230032-44239212	Disease resistance protein (TIR-NBS-LRR class), putative	−2.51	−1.76	−0.87	AT5G17680.1
Glyma06g41404	Gm06:44686411-44690459	Disease resistance protein (TIR-NBS-LRR class) family	−2.81	−3.31	−3.64	AT5G45220.1
Glyma06g41700	Gm06:44984922-44988575	Disease resistance protein (TIR-NBS-LRR class), putative	−7.63	−7.85	−7.94	AT5G17680.1
Glyma06g41880	Gm06:45152031-45155033	Disease resistance protein (TIR-NBS-LRR class), putative	−1.63	1.84	0.29	AT5G17680.1
Glyma06g46665	Gm06:49244725-49251384	Disease resistance protein (TIR-NBS-LRR class) family	−0.61	0.81	1.78	AT5G36930.2
Glyma07g07393	Gm07:6067981-6072205	Disease resistance protein (TIR-NBS-LRR class), putative	−4.23	−1.34	−1.52	AT5G17680.1
Glyma12g16450	Gm12:15730001-15734533	Disease resistance protein (TIR-NBS-LRR class), putative	−2.39	1.28	0.86	AT5G17680.1
Glyma13g03770	Gm13:3846577-3852431	Disease resistance protein (TIR-NBS-LRR class), putative	−2.38	0.65	2.81	AT5G17680.1
Glyma16g00861	Gm16:516813-521800	Disease resistance protein (TIR-NBS-LRR class) family	−3.12	−1.57	0.04	AT5G41540.1
Glyma16g25071	Gm16:28995807-29006764	Disease resistance protein (TIR-NBS-LRR class), putative	−4.12	−5.93	−11.37	AT5G17680.1
Glyma16g25120	Gm16:29058174-29061521	Disease resistance protein (TIR-NBS-LRR class), putative	−2.62	2.22	1.81	AT5G17680.1
Glyma16g32321	Gm16:35526836-35530790	Disease resistance protein (TIR-NBS-LRR class), putative	6.40	2.55	3.62	AT5G17680.1
Glyma16g33961	Gm16:36692020-36696860	Disease resistance protein (TIR-NBS-LRR class), putative	−0.24	2.06	2.19	AT5G17680.1
Glyma16g33991	Gm16:36712977-36715288	Disease resistance protein (TIR-NBS-LRR class) family	−4.80	−3.78	−3.00	AT5G36930.2
Glyma16g34086	Gm16:36774650-36776469	Disease resistance protein (TIR-NBS-LRR class), putative	0.28	2.08	3.83	AT5G17680.1
Glyma02g10320	Gm02:8186067-8188789	Heat shock protein 70	−3.81	−6.06	−4.16	AT3G12580.1
Glyma05g36600	Gm05:40426888-40430895	Heat shock protein 70 (Hsp 70) family protein	−2.39	−1.55	−1.05	AT3G12580.1
Glyma05g36620	Gm05:40443106-40447303	Heat shock protein 70	−2.33	−1.59	−0.84	AT5G42020.1
Glyma08g02940	Gm08:2029877-2033833	Heat shock protein 70 (Hsp 70) family protein	−2.63	−1.73	−1.06	AT5G02500.1
Glyma13g26890	Gm13:30070997-30076596	Heat shock protein 70B	−2.18	−3.25	−1.66	AT3G12580.1
Glyma13g29591	Gm13:32478807-32481336	Heat shock protein 70B	−1.85	−1.99	0.08	AT1G16030.1
Glyma15g09430	Gm15:6739539-6741346	Heat shock cognate protein 70-1	−3.19	−1.93	−1.03	AT5G42020.1
Glyma17g08020	Gm17:5928338-5930881	Heat shock protein 70	1.94	1.00	2.45	AT1G16030.1
Glyma18g52480	Gm18:61075241-61082432	Heat shock protein 70B	−2.17	0.12	1.14	AT5G42020.1
**Control vs. treatment in Buseok**			**C vs. 0.5 h**	**C vs. 6 h**		
Glyma05g17460	Gm05:20185056-20190951	Disease resistance protein (CC-NBS-LRR class) family	2.96	2.56		AT5G66900.1
Glyma17g21240	Gm17:20538055-20543536	Disease resistance protein (CC-NBS-LRR class) family	3.78	2.00		AT5G66900.1
Glyma03g14888	Gm03:19079981-19089860	Disease resistance protein (TIR-NBS-LRR class) family	4.46	1.16		AT5G36930.2
Glyma06g40690	Gm06:43857935-43861836	Disease resistance protein (TIR-NBS-LRR class), putative	1.55	4.35		AT5G17680.1
Glyma06g40740	Gm06:43913599-43918380	Disease resistance protein (TIR-NBS-LRR class) family	3.84	4.14		AT4G12010.1
Glyma06g41880	Gm06:45152031-45155033	Disease resistance protein (TIR-NBS-LRR class), putative	3.41	0.72		AT5G17680.1
Glyma12g16450	Gm12:15730001-15734533	Disease resistance protein (TIR-NBS-LRR class), putative	2.94	1.74		AT5G17680.1
Glyma16g25120	Gm16:29058174-29061521	Disease resistance protein (TIR-NBS-LRR class), putative	5.39	2.23		AT5G17680.1
Glyma16g33590	Gm16:36465825-36471050	Disease resistance protein (TIR-NBS-LRR class) family	4.71	3.03		AT5G36930.2

a *Dash (−) indicates uncalculated log_2_ (fold change) values due to FPKM value = 0 in Cheongja 3. A negative value represents the upregulation in Cheongja 3 for the comparison of Cheongja 3 vs. Buseok. Control, 0.5, and 6 h represent 0, 0.5, and 6 h UV-B irradiation, respectively*.

Nine members of the heat shock 70 (HSP70) protein family were differentially expressed in response to UV-B irradiation in Buseok relative to Cheongja 3, six of which were down-regulated (Table 1; Figure 3). However, there was no significant difference in the expression patterns of *HSP70* genes between control and 0.5 or 6 h UV-B treatment in Buseok or Cheongja 3.

### Stress defense signaling

Genome-wide transcript analysis in response to supplemental UV-B irradiation revealed DEGs involved in the phosphatidylinositol signaling pathway (Table 2). The phosphatidylinositol signaling pathway generates phosphatidic acid (PA) from structural phospholipids as a secondary messenger. Under UV-B stress, transcripts for phosphatidylinositol-4-phosphate 5-kinase (*PIP5K*) family protein were more abundant in Buseok than in Cheongja 3 (Table 2); this protein generates phosphatidylinositol 4,5-bisphosphate [PtdIns(4,5)P_2_] as a substrate of phospholipase C (PLC) from phosphatidylinositol 4-phosphate (Ptdins4P) in the plasma membrane (Mueller-Roeber and Pical, 2002). Also, PtdIns(4,5)P_2_ is produced from the phosphorylation of phosphatidylinositol-3,4,5-trisphosphate [PtdIns(3,4,5)P_3_] by phosphatidylinositol-3,4,5-trisphosphate 3-phosphatase and dual-specificity protein phosphatase (PTEN 2), for which one gene (Glyma20g26280) was upregulated in Buseok under UV-B irradiation (Table 2). PtdIns(4,5)P_2_ is hydrolyzed into inositol 1,4,5-trisphosphate (InsP_3_) and diacylglycerol (DAG) by phosphatidylinositol-specific PLC (PI-PLC) proteins. One DEG (Glyma18g03090) encoding PI-PLC was upregulated in Cheongja 3 under 6 h UV-B treatment relative to Buseok (Table 2). In UV-B-resistant Buseok, *PI-PLC* genes were expressed at low levels and independent of UV-B treatment. Meanwhile, two DEGs (Glyma17g08510 and Glyma06g39760) for diacylglycerol kinase (DGK) family proteins were upregulated in Buseok by UV-B stress (Table 2); these proteins convert DAG produced by PI-PLC to PA (Laxalt and Munnik, 2002). The coupling of DGK to the activation of PI-PLC may be induced in response to UV-B stress in Buseok, leading to increased levels of PA in the cells, thereby activating several downstream defense responses.

**Table 2:** **Differential expressed genes encoding five enzymes, PIP5K, PTEN 2, PI-PLC 2, DGK, and IPK2 in the phosphatidic acid signaling pathway identified by the transcriptomic comparisons of UV-B sensitive Cheongja 3 vs. resistant Buseok and control vs. UV-B treatments in Buseok**.

**Gene ID**	**Chromosome position**	**Gene definition**	**Reaction^a^**	**Log**_**2**_ **(fold change)**^b^			***A. thaliana*** **homolog**
**Cheongja 3 vs. Buseok**				**Control**	**0.5 h**	**6 h**	
Glyma10g36251	Gm10:44448484-44458944	Phosphatidylinositol-4-phosphate 5-kinase family protein (PIP5K)	PtdIns4P → PtdIns(4,5)P_2_	0.78	−0.22	1.24	AT1G34260.1
Glyma16g01590	Gm16:1161766-1173675	Phosphatidylinositol-4-phosphate 5-kinase family protein (PIP5K)	PtdIns4P → PtdIns(4,5)P_2_	0.30	2.03	1.04	AT3G14270.1
Glyma10g41000	Gm10:48199551-48208068	Phosphatidylinositol-3,4,5-trisphosphate 3-phosphatase and dual-specificity protein phosphatase (PTEN 2)	PtdIns(3,4,5)P_3_ → PtdIns(4,5)P_2_| PtdIns3P	−1.18	−1.00	−1.31	AT3G19420.1
Glyma20g26280	Gm20:35776384-35782697	Phosphatidylinositol-3,4,5-trisphosphate 3-phosphatase and dual-specificity protein phosphatase (PTEN 2)	PtdIns(3,4,5)P_3_ → PtdIns(4,5)P_2_ | PtdIns3P	−1.21	3.01	–	AT3G19420.1
Glyma18g03090	Gm18:2033237-2038137	Phosphoinositide-specific phospholipase C 2 (PI-PLC 2)	PtdIns(4,5)P_2_ → DAG + InsP_3_	−0.70	0.04	−1.56	AT3G08510.1
Glyma17g08510	Gm17:6296131-6303862	Diacylglycerol kinase1 (DGK 1)	DAG → PA	−11.43	–	1.22	AT5G07920.1
Glyma04g09290	Gm04:7455432-7461028	Inositol 1,3,4-trisphosphate 5/6-kinase family protein (IPK 2)	InsP3 → InsP6	2.38	2.75	1.93	AT4G08170.2
Glyma06g09430	Gm06:6950627-6957813	Inositol 1,3,4-trisphosphate 5/6-kinase family protein (IPK 2)	InsP3 → InsP6	0.76	1.42	–	AT4G08170.2
**Control vs. treatment in Buseok**				**C vs. 0.5 h**	**C vs. 6 h**		
Glyma06g39760	Gm06:42662480-42670185	diacylglycerol kinase 5 (DGK 5)	DAG → PA	4.69	2.33		AT2G20900.2

a Vertical bars (|) represent “either/or.”

b *Dash (−) means uncalculated log_2_ (fold change) values due to FPKM value = 0 in Cheongja3. A negative value represents the upregulation in Cheongja 3 for the comparison of Cheongja 3 vs. Buseok. Control, 0.5, and 6 h represent 0, 0.5, and 6 h UV-B irradiation, respectively*.

InsP3 generated via the hydrolysis of PI-PLC diffuses into the cytosol and is involved in the release of Ca^2+^ from intracellular stores (Ruelland et al., 2015). Owing to a lack of InsP3 receptors in plants, InsP3 is converted into the more phosphorylated forms of inositol, i.e., tetra, penta, and hexaphosphates (InsP4, InsP5, and InsP6), through further phosphorylation steps involving at least two types of inositol polyphosphate 2-kinase (IPK1) and inositol polyphosphate kinase 2 (IPK2, synonym for inositol 1,3,4-trisphosphate 5/6-kinase) (Munnik and Vermeer, 2010; Zhou et al., 2012; Sparvoli and Cominelli, 2015). Among these enzymes, Buseok exhibited enhanced expression of two *IPK2* family genes under 0.5 and 6 h UV-B irradiation compared to Cheongja 3 (Table 2). Therefore, in UV-B-resistant Buseok, the genes for four enzymes involved in the PA-dependent signaling pathway were upregulated in response to UV-B stress (Figure 3).

We also identified some DEGs encoding putative target proteins that interact with PA, including two DEGs (Glyma08g20830, Glyma19g24375) encoding protein phosphatase 2 family proteins, which were upregulated in Buseok under 0.5 h UV-B treatment (Figure 3; Supplementary Table 16); these proteins carry a PA binding motif. One example of a functionally characterized protein phosphatase targeted by PA is the protein phosphatase 2C ABI1 (ABA insensitive 1), which is bound by PA to negatively regulate ABA signaling (Zhang et al., 2004). We detected differential transcript accumulation of three genes encoding MAPKs in response to UV-B stress between Cheongja 3 and Buseok (Figure 3; Supplementary Table 16); *MAPK 19* (Glyma15g38490) and *MAPK 20* (Glyma14g03190) were upregulated in Buseok under 6 h UV-B treatment and *MAPK 3* (Glyma11g15700) was upregulated in Cheongja 3 under 0.5 h UV-B treatment (Figure 3; Supplementary Table 16). In both Buseok and Cheongja 3, however, no significant difference in *MAPK* expression was detected in the control vs. UV-B treatment comparison.

### ROS production and scavenging

Abiotic stresses including UV-B stress induce ROS production and scavenging. Four genes encoding copper/zinc superoxide dismutase (Cu/Zn SOD) family proteins, which catalyze the dismutation of superoxide anion (${\text{O}}_{2}^{•-}$) to oxygen (O_2_) and hydrogen peroxide (H_2_O_2_), were upregulated in Buseok in response to UV-B stress, while in Cheongja 3, the iron *(Fe)-SOD* gene was upregulated in response to 6 h UV-B treatment. Among the genes assigned to the GO term “oxidation reduction,” 21 cytochrome P450 (*Cyt P*_450_) family genes were identified, 12 and 9 of which were upregulated in Buseok and Cheongja 3, respectively (Figure 3; Supplementary Table 16). Six of eight *Cyt P*_450_ DEGs were downregulated in Cheongja 3 under 6 h UV-B treatment compared to the control (Supplementary Table 17). NAD(P)H-dependent electron transport involving cytochrome P450 produces ${\text{O}}_{2}^{•-}$ in the endoplasmic reticulum (Sharma et al., 2012). Genes of the Cyt P_450_ family 76 subfamily C2 and the Cyt P_450_ family 706 subfamily A are induced by UV-C irradiation (Narusaka et al., 2004). To avoid injury from ROS overproduction, ROS scavenging or detoxification is performed by antioxidative systems consisting of both nonenzymatic and enzymatic antioxidants (Sharma et al., 2012). In Buseok, we identified upregulated DEGs involved in flavonoid biosynthesis to produce phenolic compounds with antioxidant properties, such as anthocyanidin and tannin, including genes encoding flavanone-3-hydroxylase (F3H) and cinnamate-4-hydroxylase (C4H) (Supplementary Table 18). By contrast, a gene encoding the enzymatic oxidant ascorbate peroxidase was upregulated in Cheongja 3. Under 6 h UV-B treatment, genes encoding glutathione peroxidase were also upregulated only in Cheongja 3 compared to the control (Supplementary Table 17).

### RNA-seq validation by qRT-PCR

**Figure 4:**
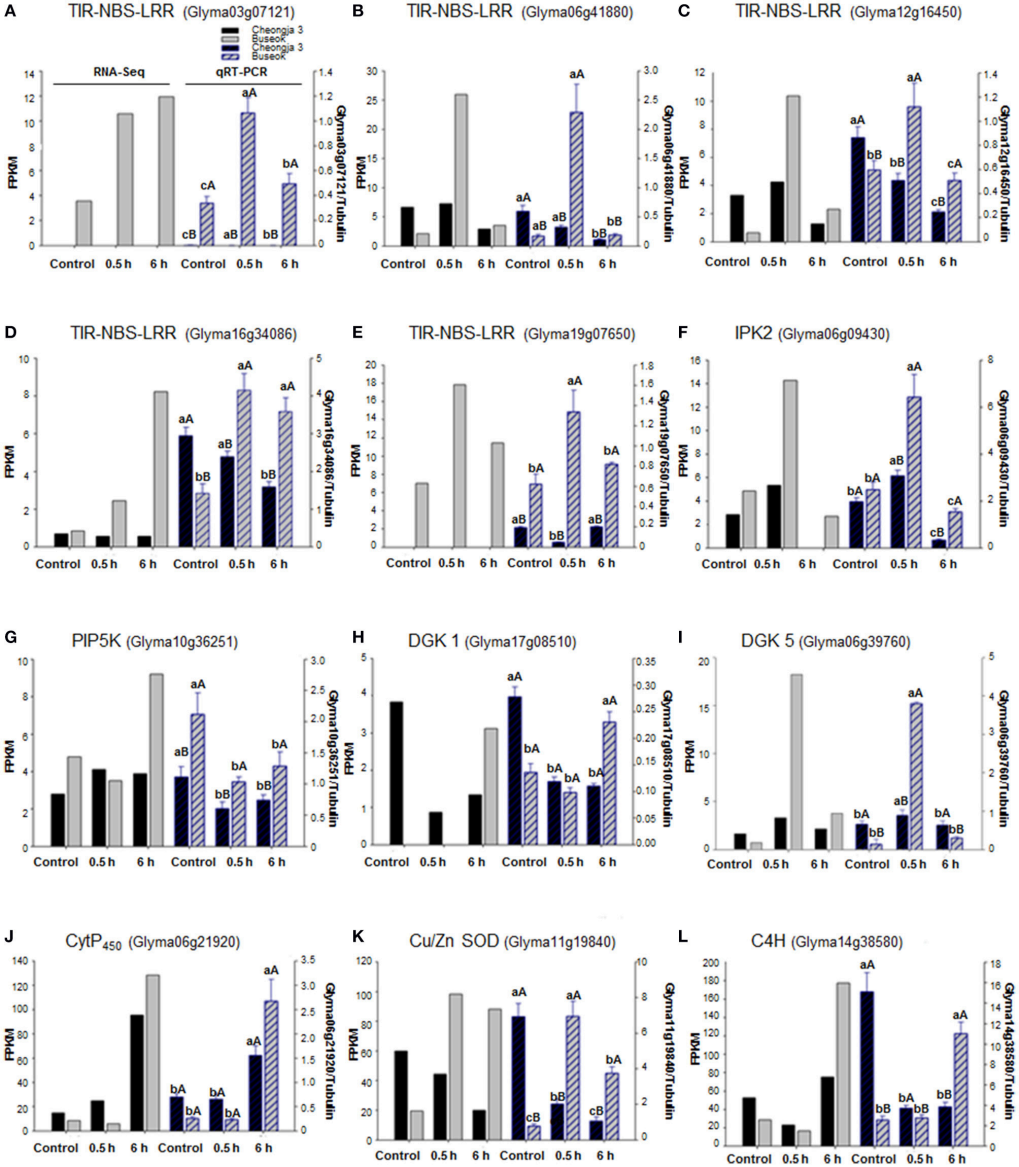
**Expression validations via qRT-PCR for UV-B related DEGs identified between UV-B-sensitive Cheongja 3 and UV-B-resistant Buseok**. Left and right Y-axes represent relative transcript abundance from RNA-seq and qRT-PCR results, respectively. Black and gray bars indicate Cheongja 3 and Buseok, respectively. Error bars represent the SE for three independent replicates. Bars with the same lower letters within a treatment indicate are not significant differences; Bars with same upper case letters within genotype indicate not significant differences by Fisher's least significant difference (LSD) test at *P* < 0.05. Control, 0.5, and 6 h on the x-axis refer to 0, 0.5, and 6 h UV-B irradiation, respectively. **(A–E)** *TIR-NBS-LRR* genes (Glyma03g07121, Glyma06g41880, Glyma12g16450, Glyma16g34086, and Glyma19g07650); **(F)** *IPK2* (Glyma06g09430); **(G)** *PIP5K* (Glyma10g36251); **(H)** *DGK 1* (Glyma17g08510); **(I)** *DGK 5* (Glyma06g39760); **(J)** *Cyt P*_450_ (Glyma06g21920); **(K)** *Cu/Zn SOD* (Glyma11g19840); **(L)** *C4H* (Glyma14g38580).

Using qRT-PCR, we confirmed that five genes (Glyma03g07121, Glyma06g41880, Glyma12g16450, Glyma16g34086, and Glyma19g07650) in the *TIR-NBS-LRR* family were upregulated in Buseok in response to 0.5 h UV-B irradiation (Figures 4A–E). The *TIR-NBS-LRRs*' expression patterns obtained by qRT-PCR also showed significantly increased transcript abundance in Buseok by UV-B treatment. Interestingly, transcript accumulation of Glyma03g07121 (TIR-NBS-LRR) was not observed in Cheongja 3 by qRT-PCR or RNA-seq. In addition, the expression profiles of individual DEGs encoding IPK2 (Glyma06g09430), PIP5K (Glyma10g36251), DGK 1 (Glyma17g08510) and DGK 5 (Glyma06g39760) in the PA signaling pathway were obtained by qRT-PCR (Figures 4F–I). Significant upregulation of *IPK2* (Glyma06g09430), *PIP5K* (Glyma10g36251) and *DGK 5* (Glyma06g39760) was observed in Buseok compared to Cheongja 3 under UV-B stress. *DGK 1* (Glyma17g08510) was significantly activated only in Buseok by 6 h UV-B irradiation relative to control. Finally, we investigated the expression patterns of three DEGs encoding *Cyt P*_450_ (Glyma06g21920), *Cu/Zn SOD* (Glyma11g19840), and *C4H* (Glyma14g38580), which are involved in ROS production and scavenging, using qRT-PCR (Figures 4J–L). *Two* genes *Cu/Zn SOD* (Glyma11g19840) and *C4H* (Glyma14g38580), except *Cyt P*_450_ (Glyma06g21920) showed significant higher expression in Buseok by both RNA-seq and qRT-PCR analyses, There was no significantly differential expression of *Cyt P450* (Glyma06g21920) between Cheongja 3 and Buseok but 6 h UV-B treatment induced upregulation of *Cyt P450* in both genotypes. All but one of the gene expression patterns measured by qRT-PCR agreed with ones analyzed by RNA-seq.

### Functional validation of UV-B stress defense signaling genes using *A. thaliana* knock-out mutants

**Figure 5:**
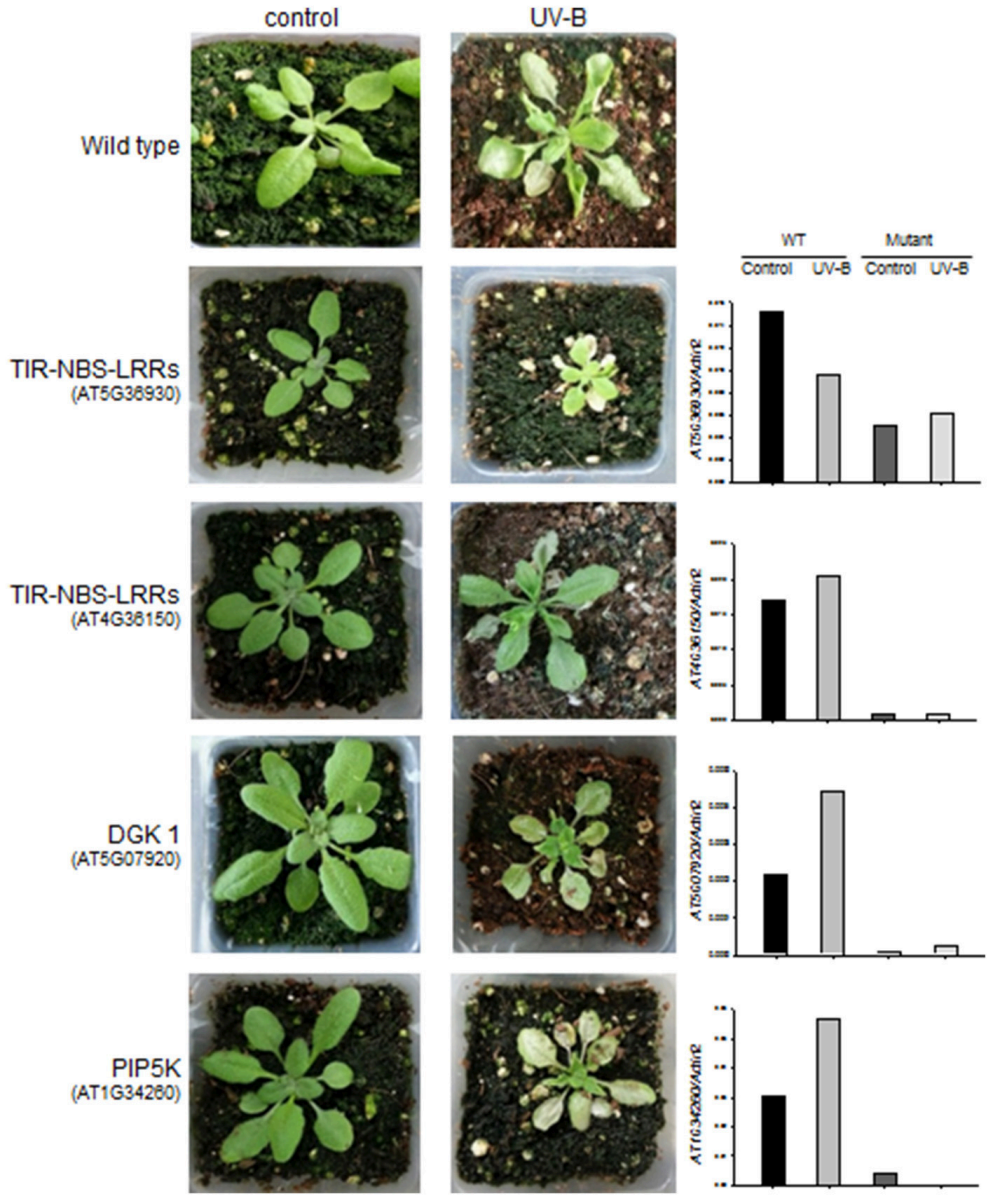
**Changes in morphology and gene expression levels of wild type and the ***A. thaliana*** knock-out mutants after 4 h UV-B treatment**. Left: morphological changes of the wild type (in the 1st row) and the four knock-out mutants (in the 2nd to 5th rows) for *TIR-NBS-LRRs* (AT5G36930 and AT4G36150), *DGK 1* (AT5G07920), and *PIP5K* (AT1G34260) between control and 4 h UV-B treatment. Right: expression levels of the target genes (*TIR-NBS-LRRs, DGK 1*, and *PIP5K*) that were measured by qRT-PCR from the wild type and the knock-out mutants under control and 6 h UV-B treatment.

We identified four *A. thaliana* knock-out mutant lines, including two for *TIR-NBS-LRR* (AT5G36930:SALK_124056C and AT4G36150: SALK_084909C), one for *DGK* (AT5G07920:SALK_033664C), and one for *PIP5K* (AT1G34260:SALK_047604C) (Figure 5). The knock-out mutant lines did not show any noticeable morphological changes compared to wild-type (Col-0) under normal growing condition, suggesting that these genes do not play vital roles in basic growth and development. However, 2 days after 4 h UV-B stress, three of the mutant lines (AT5G36930:SALK_124056C, AT5G07920:SALK_033664C, and AT1G34260:SALK_047604C) showed severe chlorosis and stagnant growth, indicating increased sensitivity to UV-B stress (Figure 5). By contrast, the wild type and the remaining mutant lines (affected in the TIR-NBS-LRR gene) exhibited continued growth despite the presence of leaf curling and slight wilting. We measured the expression patterns of the four target genes in the wild type and mutant lines in response to 4 h UV-B irradiation by qRT-PCR. In the wild type, all of the genes except AT5G36930 (*TIR-NBS-LRR*) were induced by 4 h UV-B irradiation. As expected, the expression levels of all target genes were highly reduced in the corresponding mutant lines under both control and UV-B treatment. Functional validation using the *A. thaliana* knock-out mutants revealed that some genes implicated in the phosphatidic acid signaling pathway and immune response play key roles in UV-B stress defense.

## Discussion

**Figure 6:**
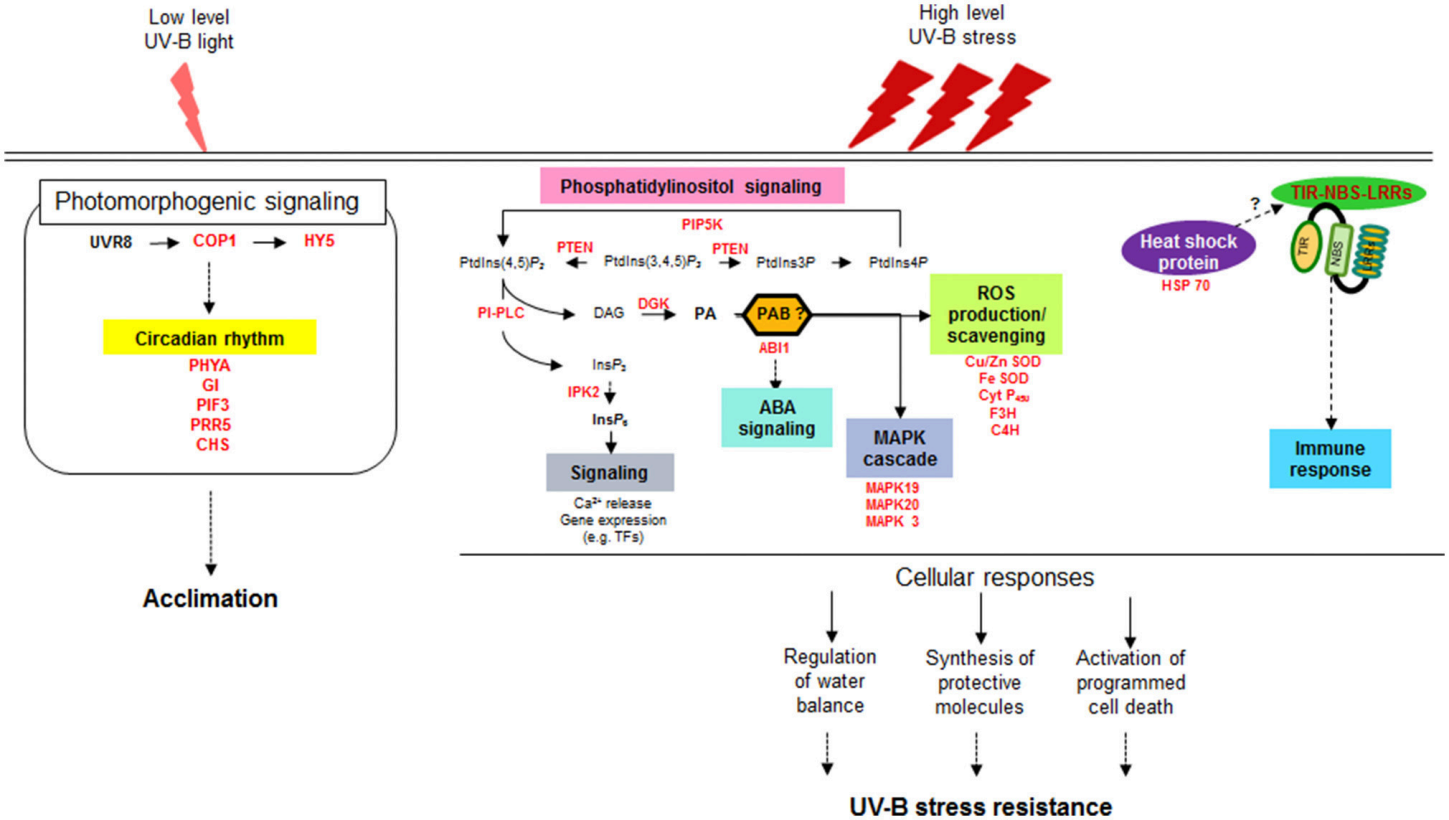
**Schematic diagram of distinct signaling pathways depending on UV-B intensity**. UVR8, UV Resistance Locus 8; COP1, Constitutively Photomorphogenic 1; HY5, Elongated Hypocotyl 5; PHYA, Phytochrome A; GI, Gigantea; PIF3, Phytochrome interacting factor 3; PRR5, Pseudo-response regulator 5; CHS, Chalcone and stilbene synthase family protein; PIP5K, phosphatidylinositol-4-phosphate 5-kinase family protein; PTEN, Phosphatidylinositol-3,4,5-trisphosphate 3-phosphatase and dual-specificity protein phosphatase; PLC, Phospholipase C; DG, Diacylglycerol; DGK, Diacylglycerol kinase; PA, Phosphatidic acid; PAB, Phosphatidic acid binding protein; ABI1, ABA insensitive phosphatase 1; IPK2, Inositol 1,3,4-trisphosphate 5/6-kinase family protein; IP6, inositol hexaphosphate; Cu/Zn SOD, Copper/Zinc superoxide dismutase; Fe- SOD, Iron-superoxide dismutase; Cyt P450, Cytochrome P450 family protein; F3H, Flavanone-3-hydroxylase; C4H, Cinnamate-4-hydroxylase; MAPK, Mitogen-activated-protein kinase; HSP 70, Heat shock protein 70; TIR-NBS-LRRs, Toll/interleukin-1 receptor nucleotide binding site leucine-rich repeat.

Two approaches are often used to investigate the responses of plants to UV-B radiation. One approach is to observe the damage and subsequent recovery of plants at specific times after UV-B irradiation (Kilian et al., 2007; Safrany et al., 2008; Biedermann and Hellmann, 2010), and the other is to investigate the accumulated responses of plants exposed to continuous UV-B treatment (Casati and Walbot, 2004; Gruber et al., 2010); both were utilized in the present study. A series of studies on the intracellular responses of maize have been conducted at different irradiation times from 5 to 90 min up to 6 h (Casati et al., 2011a,b,c), which were primarily focused on understanding plant acclimation to UV-B. In the current study, we surveyed differences in whole transcript abundance in response to supplementary UV-B exposure for 0.5 and 6 h using RNA-seq. Our transcriptome analysis provided evidence for the notion that intracellular photomorphogenic responses for adaptation to low UV-B levels are separate from defense mechanisms against UV-B stress at high fluence rates (Figure 6). While the responses to UV-B stress are considered to be mediated by signaling pathways not specific to UV-B and are also induced by other stresses, our understanding of how plants activate components of defense signaling pathways against UV-B stress remains limited.

The UV-B photoreceptor UVR8, which was isolated in *A. thaliana* (Kliebenstein et al., 2002; Favory et al., 2009), was not identified as a DEG in response to 0.5 and 6 h UV-B treatment in the current study, but other UV-B-specific photomorphogenic signaling components, including *HY5, COP1*, and *CHS*, were upregulated in UV-B-resistant Buseok compared to Cheongja 3. In addition, key genes involved in the circadian rhythm, such as *PHYTOCHROM A, GIGANTEA*, and *PHYTOCHROM INTERACTING FACTOR 3*, were upregulated by UV-B irradiation (Figure 6). Low levels of UV-B are perceived by UVR8 followed by several downstream signaling pathways (Frohnmeyer and Staiger, 2003; Heijde and Ulm, 2012). The threshold UV-B doses that initiate photomorphogenic responses are much lower than those that induce stress defense gene expression (Boccalandro et al., 2001; Brown and Jenkins, 2008; Jenkins, 2009). In the current study, it is unclear if *UVR8* expression was upregulated instantly (≤ 1–2 min) after the start of UV-B irradiation to activate downstream photomorphogenic signaling and other light signaling pathways, followed by a return to the ground state (Brown et al., 2005; Heijde and Ulm, 2012; Jenkins, 2014). However, soybean is not presumed to have another UV-B photoreceptor in addition to UVR8 for photomorphogenic responses.

Once plants recognize that irradiated UV-B fluence rates are beyond their adaptive capacity and are stressors, they immediately operate stress defense mechanisms for survival. The UV-B dosages used in our study are not low, and they represent dramatic spikes in UV-B intensity within short periods of time. Our results suggest that defense mechanisms against UV-B stress are provoked through PA-dependent signaling pathways via the sequential actions of PI-PLC and DGK, and that cell death and immunity systems also operate during this response (Figure 6). Significant advances have been made in elucidating stress-induced PA signal transduction during the past several years, as described in recent reviews (Ruelland et al., 2015; Singh et al., 2015; Hong et al., 2016; Hou et al., 2016). PA is the common product of the phospholipase D (PLD) and PLC/DGK pathways, which use different phospholipids as substrates. The PLD pathway is involved in a wide range of responses to external stresses, such as cold, salinity, drought, and pathogen attack (Hong et al., 2016). Nonetheless, we found that UV-B stress triggered only a PLC/DGK-mediated PA signaling pathway in the current study. PLC is induced by the osmotic stress caused by salt and dehydration (Darwish et al., 2009). Since UV-B stress is a complex environmental stress comprising heat and dehydration as well as excessive light exposure, our finding is in line with previous results.

UV-B-resistant Buseok showed higher transcript abundance for four key enzymes implicated in PI-PLC/DGK-mediated signaling cascades in plants (Table 2; Figures 3, 6), resulting in the production of polyphosphoinositols (membrane lipids) and inositol polyphosphates (water-soluble, IPP). These enzymes include PIP5K, PTEN2, DGK, and IPK2, exclusive of PI-PLC. In plants, the cellular levels of PtdIns(4,5)P_2_, which is produced from PtdIns4P by PIP5K and functions as a subtract for PI-PLC, are extremely low (30–100-fold lower than in mammalian cells) (Munnik and Vermeer, 2010). Nevertheless, Buseok had significantly higher levels of *PIP5K* transcript under both normal conditions (UV-B untreated) and UV-B exposure compared to Cheongja 3 (Table 2). Increased PtdIns(4,5)P_2_ levels by concurrent activation of PIP5K might lead to earlier upregulation of PI-PLC in Buseok vs. Cheongja 3 (under 0.5 h UV-B treatment), which is in agreement with the results obtained for plant tissues exposed to salt or osmotic stress (Darwish et al., 2009) as well as heat stress (Mishkind et al., 2009). In the current study, the Arabidopsis mutant defective in PIP5K showed increased sensitivity to UV-B stress compared to wild type (Figure 5). Upregulation of PI-PLC was observed only in Cheongja 3 under 6 h UV-B treatment (Table 2) and Buseok showed stable expression patterns of *PI-PLC* independent of UV-B treatment. However, the activation of DGK, which catalyzes ATP-dependent DAG phosphorylation for PA biosynthesis, is more specifically regulated in response to UV-B stress and more dependent on genotype. In Buseok, *DGK* was downregulated under control conditions and upregulated by UV-B treatment (Table 2). By contrast, in Cheongja 3, *DGK* was not significantly upregulated by UV-B treatment. Thus, the regulation of DGK is likely more critical in the PI-PLC/DGK-dependent PA signaling pathways in response to UV-B stress and appears to be transcriptionally mediated, even though the type of TF that binds to its promoter region is currently unknown. A study performed more than a decade ago revealed that a *UV-B light insensitive* (*uli*) T-DNA insertional Arabidopsis mutant, which displays hyposensitivity to low-fluence UV-B irradiation, is defective in *DGK* (Suesslin and Frohnmeyer, 2003). However, in the current study, an Arabidopsis *DGK* knock-out mutant showed increased damage to high UV-B intensity (Figure 5). Although whether DGK mediates different signaling pathways according to UV-B fluence rates is currently unclear, DGK is likely a specific component involved in UV-B-induced signal transduction in plants.

Increases in cytosolic Ca^2+^ levels in plant cells are the hallmark of stress defense responses (Singh et al., 2015). In mammalian systems, InsP_3_, the other product of PI-PLC hydrolysis of PtdIns(4,5)P_2_,releases Ca^2+^ from intracellular reservoirs by binding to ligand gated-calcium channels (Munnik and Nielsen, 2011). However, no InsP_3_ receptor has been identified in plants; instead, its multiple phosphorylated form InsP_6_, through stepwise phosphorylation by the IPP multikinases IPK2 and IPK1, is thought to stimulate increases in Ca^2+^ levels and to function as a signaling molecule itself (Munnik and Vermeer, 2010; Hou et al., 2016). In the current study, the transcript levels of IPK2, which phosphorylates Ins(1,4,5)P_3_ to Ins(1,3,4,5,6)P_5_, were higher in Buseok than in Cheongja 3 (Table 2).

PA binds to various target proteins to mediate downstream signal transduction during diverse defense responses such as ABA-mediated pathways, MAPK signaling cascades, and ROS over-accumulation (Hou et al., 2016). Among the dozens of identified effector proteins of PA in these cellular responses in plants (Ruelland et al., 2015; Hou et al., 2016), our RNA-seq analysis showed that ABI1, MAPK, and SOD were upregulated in Buseok under UV-B exposure (Figure 3). PA binding to ABI1 helps tether it to the plasma membrane where it interact with ATHB6, a negative regulator of ABA signaling involved in stomatal closure in response to drought and salinity stress (Zhang et al., 2004). PA also can bind to and activate MAPK in the response of *A. thaliana* and soybean to salt stress (Yu et al., 2010; Im et al., 2012). Based on the current results, ROS generation induced by UV-B stress is more likely dependent on SOD than on NADPH oxidase, even though PLD-derived PA binds to and activates NADPH oxidases under environmental stress (Park et al., 2004; Zhang et al., 2009).

Another interesting result of the present study is that *TIR-NBS-LRR* genes are upregulated in resistant Buseok under UV-B stress (Figure 3, Table 1; Supplementary Table 16). Plant NBS-LRR proteins can be divided into two major subfamilies based on the presence of TIR or CC motifs in their N-terminal domains (Tameling and Joosten, 2007; Göhre and Robatzek, 2008). Similarly, dozens of *TIR-NBS-LRR* genes, but no *CC-NBS-LRR*s, are differentially expressed in *A. thaliana* under heat and drought stress (Prasch and Sonnewald, 2013). A surprising role for TIR-NBS-LRRs has been proposed in the sensing of red light (Faigón-Soverna et al., 2006). A mutant of the constitutive shade avoidance (*CSA1*) gene in Arabidopsis is defective in red light-induced responses and produces a truncated protein with a structure similar to TIR.

We also observed increased accumulation of *HSP70* transcripts in response to UV-B stress in Buseok (Figure 3, Table 1; Supplementary Table 16). Indeed, in Arabidopsis, HSPs and heat shock factors are upregulated in response to pathogen infection, abiotic stresses including UV, and wounding (Swindell et al., 2007). HSPs including HSP90 are thought to regulate the function of NBS-LRR (Belkhadir et al., 2004). Similarly, in soybean, HSP70 is upregulated under high temperature stress (Ahsan et al., 2010). Therefore, the highly expressed TIR-NBS-LRR proteins and HSPs under UV-B stress likely play important roles in the UV-B resistance response. Further studies on which and how genetic factors activate the transcription of *TIR-NBS-LRR* and *HSP* genes will be valuable for breeding of UV-B resistant soybean cultivars.

Taken with the previous reports together, a valid assumption as shown in Figure 6 can be drawn from our transcriptomic results, which is that high fluence rates of UV-B triggers the multiple defense pathways that are overlap with defense signaling pathways against other stress but independent of UVR8-mediated UV-B pathways at ambient levels. The second signal messenger PA, generated by the reaction of PI-PLC and DGK, induces the expression of genes characteristic of stress responses such as ABA signaling, MAPK signaling cascades and ROS accumulation. Meantime (or subsequently), the genes for TIR-NBS-LRRs and HSPs are also activated under high dose UV-B, involved in programmed cell death and immune response. Orchestration of these multiple defense pathways leads to the regulation of water balance and synthesis of protective molecules, resulting in showing UV-B stress resistance in plants.

The present study provides comprehensive insights into defense signaling pathways against high-intensity UV-B stress, from signal transduction by second messengers to downstream defense-related gene expression (Figure 6). It is currently likely indisputable that the generation of PPI-based signaling molecules such as PA and IP_6_ is the primary event in the signaling cascades from stress awareness to defensive metabolism. Based on our findings, further studies should be performed investigating how the key enzymes involved in PA signaling pathways are upregulated under UV-B stress and how the derived signaling molecules are integrated into downstream pathways, together with the identification of TFs specific to UV-B stress. Such studies will provide essential information for breeding resistant soybean genotypes that survive under high-intensity UV-B stress and can adapt to other adverse conditions for sustainable productivity in the future.

## Author contributions

MY performed all experiments and prepared the manuscript. MK participated in work design and data interpretation. SS performed RNA-seq bioinformatic analyses. KK provided the plant materials. JH performed statistical analysis of qRT-PCR data. JS and SK revised the manuscript. SL initiated and coordinated the project.

### Conflict of interest statement

The authors declare that the research was conducted in the absence of any commercial or financial relationships that could be construed as a potential conflict of interest.
