# Supplementary material for: Transcriptomic Profiling of Soybean in Response to High-Intensity UV-B Irradiation Reveals Stress Defense Signaling
Source: Front Plant Sci. 2016 Dec 19;7:1917. doi: 10.3389/fpls.2016.01917 (PMC5165247; doi:10.3389/fpls.2016.01917)
Supplement: Supplementary file 3 [file Image1.PDF]

Supplementary Material

Transcriptomic profiling of soybean in response to high-intensity UV-B irradiation reveals stress defense signaling

Min Young Yoon[1], Moon Young Kim[1,2], Sangrae Shim[1], Kyung Do Kim[3], Jungmin Ha[1,2], Jin Hee Shin[3], Sungtaeg Kang[4], Suk-Ha Lee[1,2*]

*** Correspondence:** Suk-Ha Lee (sukhalee@snu.ac.kr)

1. Supplementary Figures and Tables

1.1 Supplementary Figures

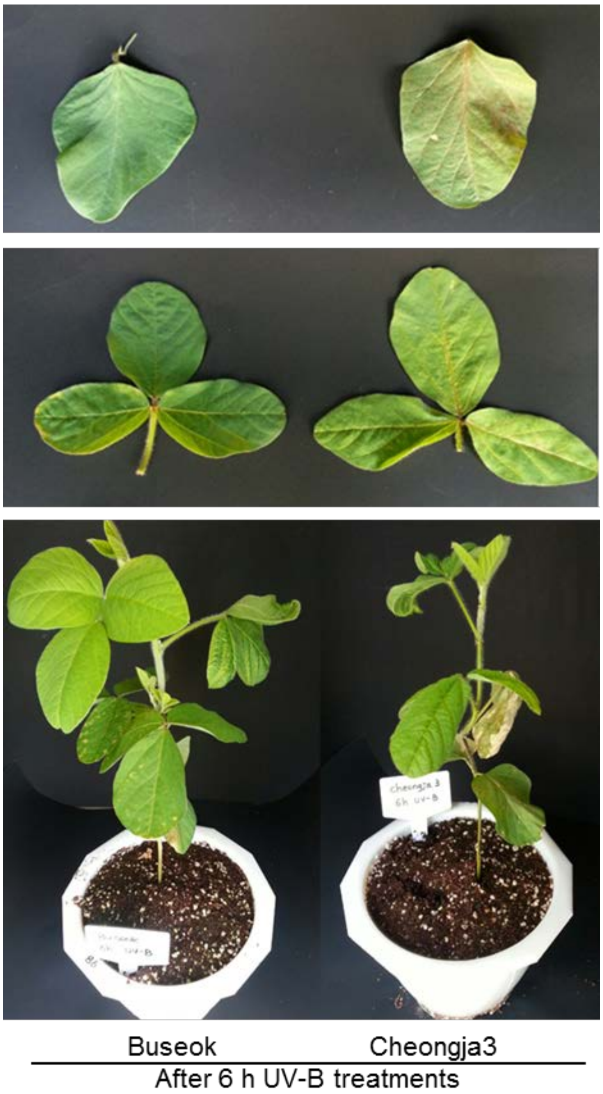

Figure S1 Morphological changes of UV-B-resistant Buseok and UV-B-sensitive Cheongja 3 after 6 h UV-B irradiation. Buseok showed fewer damaged leaves and vigorous growth, whereas Cheongja 3 showed more yellow leaves with red spots, ultimately leading to defoliation.

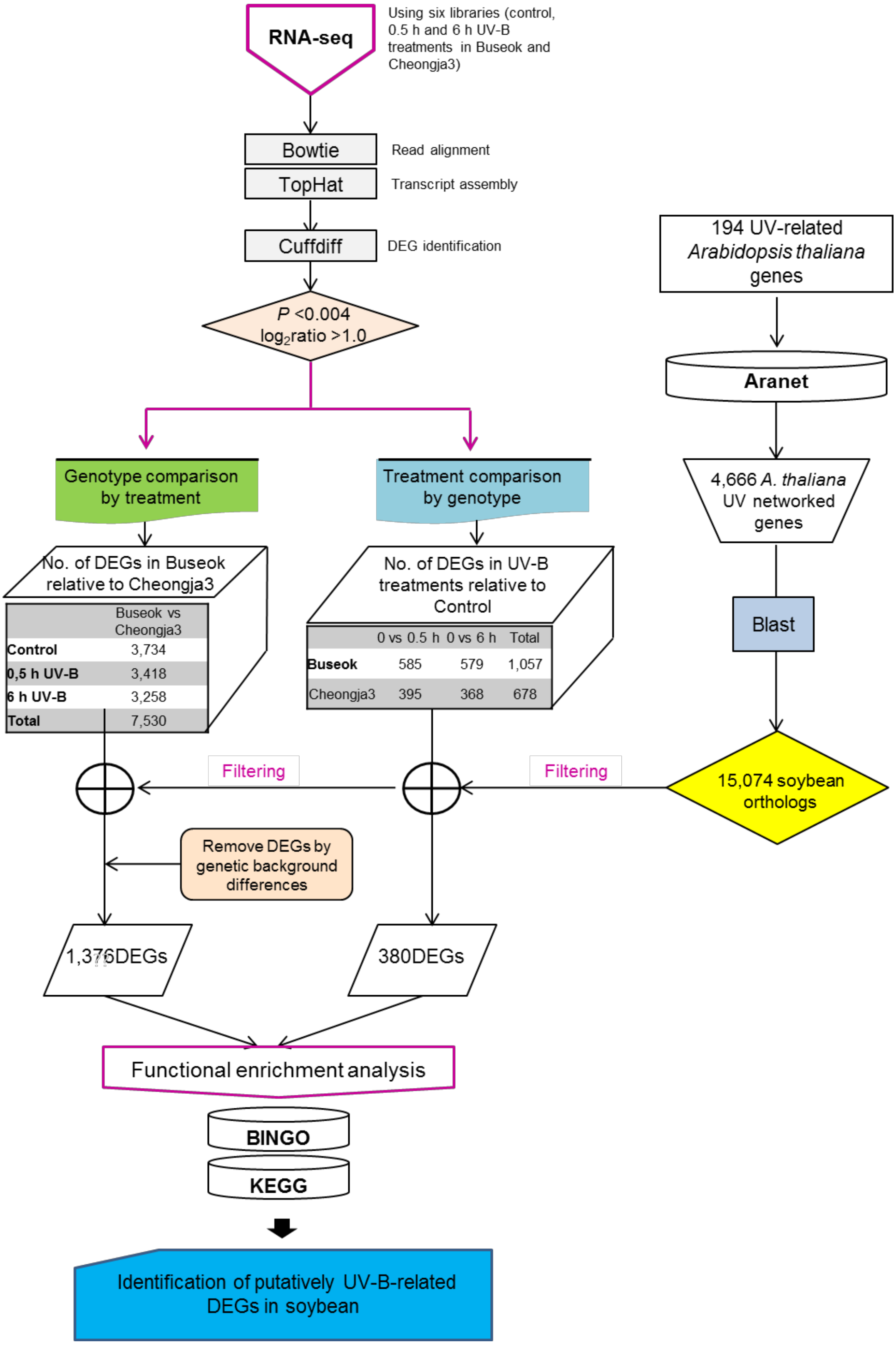

Figure S2 The workflow of two-way transcriptomic comparisons. Genotype comparison: the comparisons between Cheongja3 and Buseok under 0 (control), 0.5, and 6 h UV-B treatments. Treatment comparison: the comparisons between control and 0.5 & 6 h UV-B treatments in Buseok and Cheongja 3.

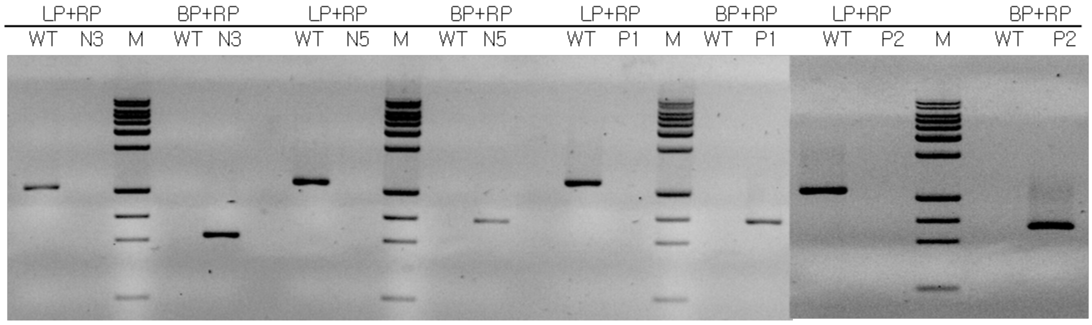

Figure S3 Validation of Arabidopsis knock-out mutants for the target genes by RT-PCR. LP; left primer, RP: right primer; BP: border primer; WT: wild type (Col-0); N3: *TIR-NBS-LRRs* (AT5G36930.2); N5: *TIR-NBS-LRRs* (AT4G36150.1); P1: diacylglycerol kinase1 (*DGK 1*) (AT5G07920.1); P2: Phosphatidylinositol-4-phosphate-5-kinase (*PIP5K*)

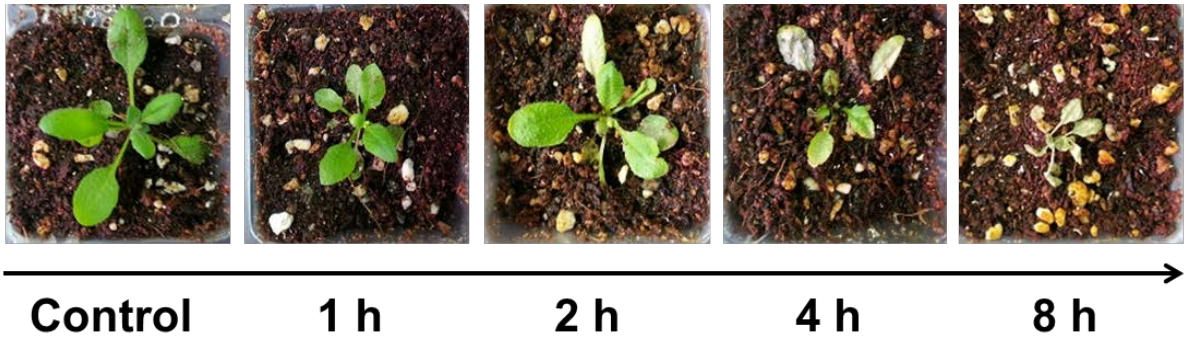

Figure S4 Morphological changes of Arabidopsis wild type (Col-0) under UV-B irradiation for 1, 2, 4 and 8 h.

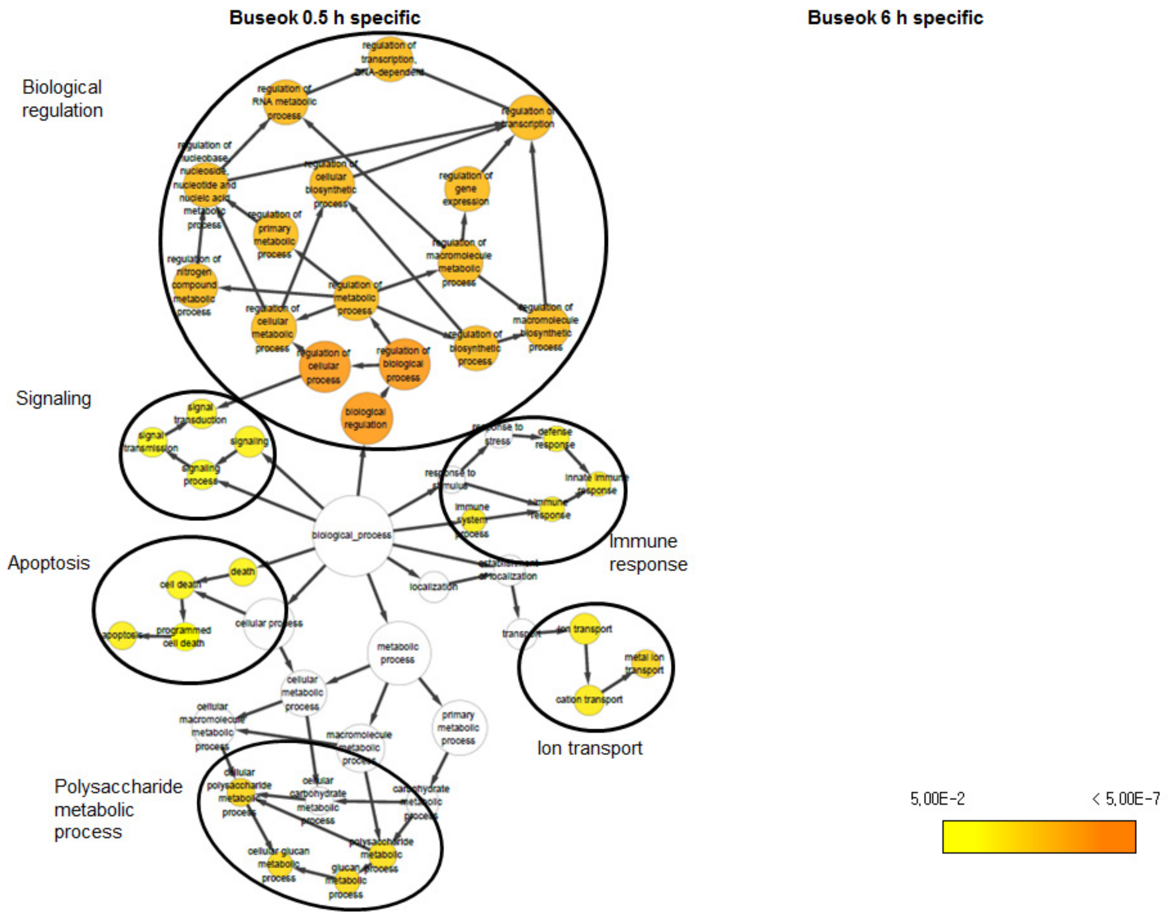

Figure S5 GO term overrepresentation analysis of the DEGs identified from the comparison of control vs 0.5 & 6 h UV-B treatments in Buseok.

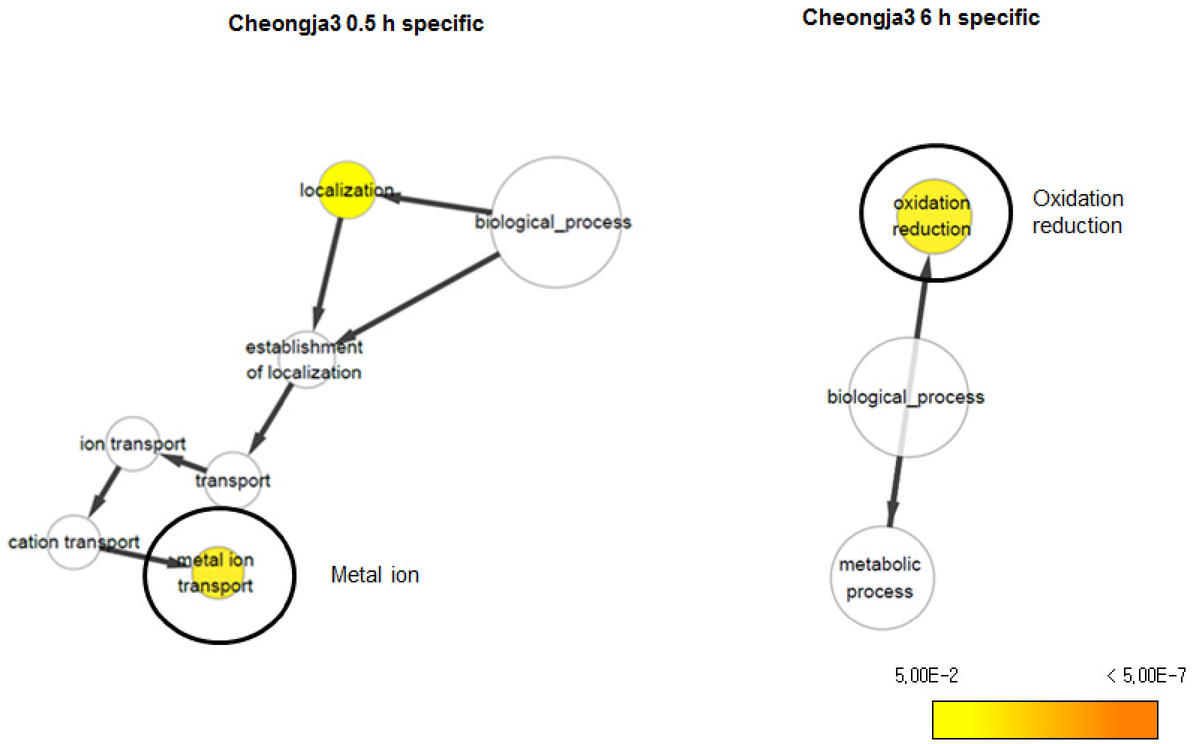

Figure S6 GO term overrepresentation analysis of the DEGs identified from the comparison of control vs 0.5 & 6 h UV-B treatments in Cheongja 3.

2. Supplementary Tables

Supplementary Table 1-2 Supplementary Table 1-2 **_**Frontiers.xlsx

Supplementary Table 1 List of 194 UV-B related genes in *A. thaliana* and their orthologous genes in soybean

Supplementary Table 2 List of 4,666 Arabidopsis genes that were predicted to respond directly or indirectly to UV-B by Aranet

Supplementary Table 3 The DEGs that were validated by qRT-PCR and their qRT-PCR primer sequences.

Category	Gene ID	Chromosome position	Gene definition	*A.thaliana* homolog	Primer
TIR-NBS-LRRs	Glyma03g07121	Gm03:7458626-7491220	disease resistance protein (TIR-NBS-LRR class), putative	AT5G17680.1	F: GCTGGGGTTAGCAATTGAAG<br>R: GCAAACACCATAGCGAACCT
	Glyma06g41880	Gm06:45152031-45155033	disease resistance protein (TIR-NBS-LRR class), putative	AT5G17680.1	F: TGGGATGGAAATGCCTTAAA<br>R: GGTGGACAATGGAAAGGATG
	Glyma12g16450	Gm12:15730001-15734533	disease resistance protein (TIR-NBS-LRR class), putative	AT5G17680.1	F: GGGATGCAGAGCTTTTGAAG<br>R: TACCACAAACGTGGAGACCA
	Glyma16g34086	Gm16:36774650-36776469	Disease resistance protein (TIR-NBS-LRR class) family	AT5G36930.2	F: AGGGAAGGGCTGTTGGTTAT<br>R: TCCTGCAGCTTCTCCATCTT
	Glyma19g07650	Gm19:9152696-9157598	Disease resistance protein (TIR-NBS-LRR class) family	AT5G36930.2	F: CAAAAGGGAGCAGCTACAGG<br>R: TTCGTTCAACACCGTGACAT
Phosphatidylinositol signaling	Glyma06g09430	Gm06:6950627-6957813	Inositol 1,3,4-trisphosphate 5/6-kinase family protein (*IPK 2*)	AT4G08170.2	F: AAAGGTCGTTAGGCGGTTTT<br>R: TCCAAATCAGCATCATCTGC
	Glyma10g36251	Gm10:44448484-44458944	Phosphatidylinositol-4-phosphate 5-kinase family protein (*PIP5K*)	AT1G34260.1	F: TATGACCCCCTCATGGTGAT<br>R: AGCTCCATCAGCAGCAGAAT
	Glyma17g08510	Gm17:6296131-6303862	diacylglycerol kinase1 (*DGK 1*)	AT5G07920.1	F: CCGTTGCTGTACTTGTGGTG<br>R: CAACGAACAGTCCATTGGTG
	Glyma06g39760	Gm06:42662480-42670185	diacylglycerol kinase5 (*DGK 5*)	AT2G20900.2	F: CAAGGATGGTTCTTTGGTTCTC<br>R: TGGAGATCTTCCCAATGACC
Oxidation reduction	Glyma06g21920	Gm06:18534605-18541507	Cytochrome P450 superfamily protein (*Cyt P450*)	AT5G07990.1	F: TGACCCATTGGAGTTTAGGC<br>R: CGTCCAGCACCAAAAGGTAT
	Glyma11g19840	Gm11:16619909-16623705	copper/zinc superoxide dismutase 2 (*Cu/Zn SOD 2*)	AT2G28190.1	F: GCTCACTCCCCAATCCATAA<br>R: CTTCCACCAGCATTTCCAGT
	Glyma14g38580	Gm14:47721241-47725350	cinnamate-4-hydroxylase (*C4H*)	AT2G30490.1	F: CACCAAGTGACTGAGCCAGA<br>R: ATGTGTGGGACAAGGAGAGG

Supplementary Table 4 RT-PCR primer sequences for identification of homozygous *A. thaliana* knock-out mutants.

Target	Gene definition	*A.thaliana* homolog	Left primer (LP)	Right primer (RP)	LP+RP Product	BP+RP product
NBS-LRRs	N3: disease resistance protein (TIR-NBS-LRR class) family	AT5G36930.2	TCTGAATTCACTAGCCATGGC	ATTCGCTTGTAGCTTTTTCCC	1050	460-760
	N5: disease resistance protein (TIR-NBS-LRR class) family	AT4G36150.1	TTGGTTCCTCGTACACCAAAC	GCAGTTCCAGAATTCCTAGGG	1112	580-880
Phosphatidylinositol signaling	P1: diacylglycerol kinase1	AT5G07920.1	GCAATCAAAAGTTTAGTCGAAACTG	AGAGATCTAGAAAGCGACCCG	1148	568-868
	P2: Phosphatidylinositol-4-phospate-5-kinase	AT1G34260.1	TTCAACCAATGGACAAGAAGC	GAACATCGTCTTCGCCATTAG	1106	541-841

>BP_LBb1.3 ATTTTGCCGATTTCGGAAC

Supplementary Table 5 qRT-PCR primer sequences to measure expression levels of the target genes in *A.thaliana* knock-out mutants.

Target	Gene definition	A.thaliana homolog	TAIR ID	Left primer	Right primer	Product size
NBS-LRRs	Disease resistance protein (TIR-NBS-LRRs class) family	AT5G36930.2	SALK_124056C	AGCGAAATCTGTTGGCAAAT	GATGAACTGATCCCGGAAGA	96
	Disease resistance protein (TIR-NBS-LRRs class) family	AT4G36150.1	SALK_084909C	GGTGTACGAGGAACCAAACC	ATGCTCTCTCCAACGGACAT	87
Phosphatidylinositol signaling pathway	Diacylglycerol kinase 1	AT5G07920.1	SALK_033664C	AACAACCACGTCTCCGTCTT	CGAGGAGAAGGATTGTGAGG	80
	Phosphatidylinositol-4-phosphate-5-kinase (PIP5K)	AT1G34260.1	SALK_047604C	TCGTTTCACAGCCACTTCTG	TAACGTAAAGCGGGGATTTG	92

Supplementary Table 6 Summary of RNA-seq data derived from Illumina Hiseq2000
100 bp paired-end RNA sequencing

Sample	Number of reads	Sum of read length	Number of properly mapped read	Total mapped depth	Total mapped position	Mapping coverage
Buseok-control	47108676	4710867600	36206146 (76.86%)	4250337952	183775226	23.1
Buseok-0.5 h	50348574	5034857400	39428414 (78.31%)	4721972620	195522924	24.1
Buseok-6 h	55178917	5517891700	43393678 (78.64%)	5343938022	200021200	26.7
Cheongja3-control	50972823	5097282300	39156576 (76.82%)	4756405618	195574728	24.3
Cheongja3-0.5 h	62332947	6233294700	49042114 (78.68%)	5853644374	203376470	28.8
Cheongja3-6 h	52252434	5225243400	40819278 (78.12%)	4989506691	200589198	24.9

Insert size: 500 bp
Six samples in 1 lane

Mapping reference: Gmax_109.Chloro.fa (975Mb)
Reference CDS length: 87 Mb

Supplementary Table 7-9 Supplementary Table 7-9_Frontiers.xlsx

Supplementary Table 7 List of the DEGs identified by the comparison of control vs UV-B treatments in UV-B tolerant Buseok

Supplementary Table 8 List of the DEGs identified by the comparison of control vs UV-B treatments in UV-B sensitive Cheongja 3

Supplementary Table 9 List of the DEGs identified by the comparison of Cheongja 3 vs Buseok under 0.5 and 6 h UV-B treatments

Supplementary Table 10 Over-represented GO terms of the DEGs identified by the comparison between Cheongja 3 and Buseok under 0.5 and 6 h UV-B treatments.

**GO ID**	**Description**	**p-value**	**Input number**	**Background number**
0002376	Immune system process	7.87E-14	31	149
0006955	Immune response	7.87E-14	31	149
0045087	Innate immune response	7.87E-14	31	149
0006952	Defense response	1.88E-09	31	217
0016265	Death	6.60E-06	36	389
0008219	Cell death	6.60E-06	36	389
0012501	Programmed cell death	7.94E-07	36	355
0006915	Apoptosis	7.94E-07	36	355
0032502	Developmental process	3.31E-04	10	65
0048869	Cellular developmental process	1.56E-05	9	37
0048856	Anatomical structure develoment	1.22E-05	9	36
0009653	Anatomical structure morphogenesis	1.22E-05	9	36
0032989	Cellular component morphogenesis	1.22E-05	9	36
0000902	Cell morphogenesis	1.22E-05	9	36
0023052	Signaling	4.86E-04	43	607
0023046	Signaling process	1.65E-04	43	65
0023060	Signal transmission	1.65E-04	43	577
0007165	Signal transduction	2.73E-05	41	499
0008152	Metabolic process	2.79E-05	508	11035
0055114	Oxidation reduction	8.71E-05	128	2237
0006801	Superoxide metabolic process	3.80E-06	6	12
0006800	Oxygen and reactive oxygen species metabolic process	3.80E-06	6	12
0009765	Photosynthesis, Light harvesting	2.53E-03	6	34
0006560	Proline metabolic process	1.31E-03	3	6
0044283	Small molecule biosynthetic process	8.61E-04	39	550
0016053	Organic acid biosynthesis process	5.41E-04	25	292
0046394	Carboxylic acid biosynthetic process	5.41E-04	25	292
0008610	Lipid biosynthetic process	1.70E-03	29	387
0006633	Fatty acid biosynthetic process	1.80E-03	11	94
0046173	Poyol biosynthetic process	6.75E-04	3	5
0006021	Inositol biosynthetic process	6.75E-04	3	5
Total			690	16,571

Supplementary Table 11 Over-represented GO terms of the DEGs identified by the comparison between control and 0.5 h UV-B treatment in Buseok.

GO ID	Description	p-value	Input number	Background number
0050794	Regulation of cellular process	2.37E-08	59	2739
0065007	Biological regulation	3.09E-08	61	2903
0050789	Regulation of biological process	3.68E-08	59	2772
0051252	Regulation of RNA metabolic process	2.06E-06	42	1883
0006355	Regulation of transcription, DNA-dependent	2.06E-06	42	1883
0045449	Regulation of transcription	2.15E-06	42	1886
0031326	Regulation of cellular biosynthetic process	2.74E-06	42	1904
0009889	Regulation of biosynthetic process	2.74E-06	42	1904
0010556	Regulation of macromolecule biosynthetic process	2.74E-06	42	1904
0010468	Regulation of gene expression	3.05E-06	42	1912
0060255	Regulation of macromolecule metabolic process	3.49E-06	42	1922
0031323	Regulation of cellular metabolic process	4.30E-06	43	2007
0019222	Regulation of metabolic process	5.27E-06	43	2023
0051171	Regulation of nitrogen compound metabolic process	5.37E-06	42	1955
0019219	Regulation of nucleobase, nucleoside, nucleotide and nucleic acid metabolic process	5.37E-06	42	1955
0080090	Regulation of primary metabolic process	6.60E-06	42	1971
0030001	Metal ion transport	8.14E-05	11	252
0044264	Cellular polysaccharide metabolic process	8.43E-05	11	253
0006073	Cellular glucan metabolic process	1.38E-04	8	142
0044042	Glucan metabolic process	1.45E-04	8	143
0005976	Polysaccharide metabolic process	2.32E-04	11	284
0006812	Cation transport	7.73E-04	14	491
0006811	Ion transport	1.02E-03	15	564
0045087	Innate immune response	1.07E-03	7	149
0006955	Immune response	1.07E-03	7	149
0002376	Immune system process	1.07E-03	7	149
0006915	Apoptosis	1.47E-03	11	355
0012501	Programmed cell death	1.47E-03	11	355
0023052	Signaling	2.10E-03	15	607
0006952	Defense response	2.26E-03	8	217
0007165	Signal transduction	2.63E-03	13	499
0016265	Death	3.00E-03	11	389
0008219	Cell death	3.00E-03	11	389
0023060	Signal transmission	8.73E-03	13	577
0023046	Signaling process	8.73E-03	13	577
Total			176	16,571

Supplementary Table 12 Over-represented GO terms of the DEGs identified by the comparison between control and 0.5 & 6 h UV-B treatments in Cheongja 3.

GO ID	Description	p-value	Input number	Background number
**C vs 0.5 h**				
0030001	Metal ion	4.60E-05	9	252
**C vs 6 h**				
0055114	Oxidation reduction	8.02E-05	26	2237

Supplementary Table 13 Over-represented KEGG pathways of the DEGs identified by the comparison between Cheongja 3 and Buseok under 0.5 and 6 h UV-B treatments.

Pathway category	Pathway name	Number of sequence	Number of enzyme	Pathway ID
Environmental Information Processing; Signal transduction	Phosphatidylinositol signaling system	6	5	gmx04070
Environmental Information Processing; Signal transduction	mTOR signaling pathway	3	1	map04150
Immune system	T cell receptor signaling pathway	8	2	map04660
Amino acid metabolism	Phenylalanine metabolism	29	6	gmx00360
Amino acid metabolism	Arginine and proline metabolism	23	8	gmx00330
Amino acid metabolism	Alanine, aspartate and glutamate metabolism	21	8	gmx00250
Amino acid metabolism	Cysteine and methionine metabolism	21	7	gmx00270
Amino acid metabolism	Glycine, serine and threonine metabolism	19	9	gmx00260
Amino acid metabolism	Phenylalanine, tyrosine and tryptophan biosynthesis	14	7	gmx00400
Amino acid metabolism	Tyrosine metabolism	14	7	gmx00350
Amino acid metabolism	Tryptophan metabolism	9	3	gmx00380
Amino acid metabolism	Histidine metabolism	9	2	gmx00340
Amino acid metabolism	Lysine degradation	7	3	gmx00310
Amino acid metabolism	Lysine biosynthesis	5	2	gmx00300
Amino acid metabolism	Valine, leucine and isoleucine biosynthesis	1	1	gmx00290
Biosynthesis of other secondary metabolites	Phenylpropanoid biosynthesis	18	7	gmx00940
Biosynthesis of other secondary metabolites	Flavonoid biosynthesis	30	7	gmx00941
Biosynthesis of other secondary metabolites	Tropane, piperidine and pyridine alkaloid biosynthesis	13	5	gmx00960
Biosynthesis of other secondary metabolites	Novobiocin biosynthesis	11	4	map00401
Biosynthesis of other secondary metabolites	Isoquinoline alkaloid biosynthesis	11	3	gmx00950
Biosynthesis of other secondary metabolites	Flavone and flavonol biosynthesis	7	2	gmx00944
Biosynthesis of other secondary metabolites	Streptomycin biosynthesis	5	3	map00521

Biosynthesis of other secondary metabolites	Isoflavonoid biosynthesis	4	2	gmx00943
Biosynthesis of other secondary metabolites	Caffeine metabolism	4	1	gmx00232
Biosynthesis of other secondary metabolites	Anthocyanin biosynthesis	2	1	map00942
Biosynthesis of other secondary metabolites	Stilbenoid, diarylheptanoid and gingerol biosynthesis	2	1	gmx00945
Biosynthesis of other secondary metabolites	Butirosin and neomycin biosynthesis	2	2	map00524
Carbohydrate metabolism	Starch and sucrose metabolism	45	17	gmx00500
Carbohydrate metabolism	Glycolysis / Gluconeogenesis	22	11	gmx00010
Carbohydrate metabolism	Amino sugar and nucleotide sugar metabolism	18	14	gmx00520
Carbohydrate metabolism	Glyoxylate and dicarboxylate metabolism	15	5	gmx00630
Carbohydrate metabolism	Galactose metabolism	12	6	gmx00052
Carbohydrate metabolism	Fructose and mannose metabolism	11	5	gmx00051
Carbohydrate metabolism	Pentose and glucuronate interconversions	10	7	gmx00040
Carbohydrate metabolism	Pentose phosphate pathway	10	8	gmx00030
Carbohydrate metabolism	Valine, leucine and isoleucine degradation	9	2	gmx00280
Carbohydrate metabolism	Pyruvate metabolism	9	2	gmx00620
Carbohydrate metabolism	Butanoate metabolism	8	5	gmx00650
Carbohydrate metabolism	Propanoate metabolism	8	4	gmx00640
Carbohydrate metabolism	Inositol phosphate metabolism	7	5	gmx00562
Carbohydrate metabolism	Ascorbate and aldarate metabolism	7	3	gmx00053
Carbohydrate metabolism	Citrate cycle (TCA cycle)	5	2	gmx00020
Energy metabolism	Carbon fixation in photosynthetic organisms	25	7	gmx00710
Energy metabolism	Nitrogen metabolism	24	11	gmx00910
Energy metabolism	Methane metabolism	18	11	map00680
Energy metabolism	Oxidative phosphorylation	11	3	gmx00190
Energy metabolism	Sulfur metabolism	5	2	gmx00920
Energy metabolism	Carbon fixation pathways in prokaryotes	2	2	map00720
Energy metabolism	Photosynthesis	1	1	gmx00195

Genetic information processing; Translation	Aminoacyl-tRNA biosynthesis	4	4	gmx00970
Glycan biosynthesis and metabolism	Glycosylphosphatidylinositol(GPI)-anchor biosynthesis	1	1	gmx00563
Lipid metabolism	Glycerolipid metabolism	29	5	gmx00561
Lipid metabolism	Fatty acid metabolism	11	5	gmx01212
Lipid metabolism	Biosynthesis of unsaturated fatty acid	9	4	gmx01040
Lipid metabolism	Fatty acid elongation	8	2	gmx00062
Lipid metabolism	Steroid hormone biosynthesis	8	3	map00140
Lipid metabolism	alpha-Linolenic acid metabolism	7	4	gmx00592
Lipid metabolism	Arachidonic acid metabolism	7	4	gmx00590
Lipid metabolism	Glycerophospholipid metabolism	7	5	gmx00564
Lipid metabolism	Linoleic acid metabolism	6	3	gmx00591
Lipid metabolism	Sphingolipid metabolism	6	2	gmx00600
Lipid metabolism	Fatty acid biosynthesis	5	2	gmx00061
Lipid metabolism	Synthesis and degradation of ketone bodies	5	3	gmx00072
Lipid metabolism	Ether lipid metabolism	4	1	gmx00565
Lipid metabolism	Sesquiterpenoid and triterpenoid biosynthesis	3	1	gmx00909
Lipid metabolism	Fatty acid degradation	2	2	gmx00071
Lipid metabolism	Steroid biosynthesis	1	1	gmx00100
Metabolism of cofactors and vitamins	Ubiquinone and other terpenoid-quinone biosynthesis	15	4	gmx00130
Metabolism of cofactors and vitamins	Porphyrin and chlorophyll metabolism	12	5	gmx00860
Metabolism of cofactors and vitamins	Thiamine metabolism	11	1	gmx00730
Metabolism of cofactors and vitamins	One carbon pool by folate	11	5	gmx00670
Metabolism of cofactors and vitamins	Retinol metabolism	8	4	
Metabolism of cofactors and vitamins	Biotin metabolism	5	2	gmx00780
Metabolism of cofactors and vitamins	Vitamin B6 metabolism	4	3	gmx00750
Metabolism of cofactors and vitamins	Riboflavin metabolism	3	3	gmx00740
Metabolism of cofactors and vitamins	Folate biosynthesis	2	1	gmx00790

Metabolism of cofactors and vitamins	Lipoic acid metabolism	1	1	gmx00785
Metabolism of cofactors and vitamins	Pantothenate and CoA biosynthesis	1	1	map00770
Metabolism of other amino acids	Glutathione metabolism	13	7	gmx00480
Metabolism of other amino acids	beta-Alanine metabolism	4	3	gmx00410
Metabolism of other amino acids	Selenocompound metabolism	4	2	
Metabolism of other amino acids	Cyanoamino acid metabolism	3	1	gmx00460
Metabolism of terpenoids and polyketides	Polyketide sugar unit biosynthesis	5	3	map00523
Metabolism of terpenoids and polyketides	Limonene and pinene degradation	4	2	gmx00903
Metabolism of terpenoids and polyketides	Geraniol degradation	4	2	map00281
Metabolism of terpenoids and polyketides	Terpenoid backbone biosynthesis	3	4	gmx00900
Metabolism of terpenoids and polyketides	Monoterpenoid biosynthesis	2	2	gmx00902
Metabolism of terpenoids and polyketides	Zeatin biosynthesis	2	2	gmx00908
Metabolism of terpenoids and polyketides	Biosynthesis of vancomycin group antibiotics	2	1	map01055
Nucleotide metabolism	Purine metabolism	38	9	gmx00230
Nucleotide metabolism	Pyrimidine metabolism	10	6	gmx00240
Xenobiotics biodegradation and metabolism	Drug metabolism - cytochrome P450	10	3	map00982
Xenobiotic biodegradation and metabolism	Benzoate degradation	7	3	map00362
Xenobiotics biodegradation and metabolism	Aminobenzoate degradation	5	4	map00627
Xenobiotic biodegradation and metabolism	Steroid degradation	4	2	map00984
Xenobiotics biodegradation and metabolism	Caprolactam degradation	3	1	map00930
Xenobiotics biodegradation and metabolism	Chlorocyclohexane and chlorobenzene degradation	2	1	map00361
Xenobiotics biodegradation and metabolism	Chloroalkane and chloroalkene degradation	2	2	map00625
Xenobiotics biodegradation and metabolism	Drug metabolism - other enzymes	2	2	map00983
Xenobiotics biodegradation and metabolism	Naphthalene degradation	1	1	map00626
Xenobiotics biodegradation and metabolism	Styrene degradation	1	1	map00643

Supplementary Table 14 Over-represented KEGG pathways of the DEGs identified by the comparison between control and UV-B treatments in Buseok.

Pathway category	Pathway	Number of sequence	Number of enzyme	Pathway ID
Amino acid metabolism	Alanine, aspartate and glutamate metabolism	1	1	gmx00250
Amino acid metabolism	Phenylalanine metabolism	1	1	gmx00360
Amino acid metabolism	Tryptophan metabolism	1	1	gmx00380
Biosynthesis of other secondary metabolites	Phenylpropanoid biosynthesis	2	2	gmx00940
Biosynthesis of other secondary metabolites	Caffeine metabolism	1	1	gmx00232
Biosynthesis of other secondary metabolites	Flavonoid biosynthesis	1	1	gmx00943
Biosynthesis of other secondary metabolites	Flavonoid biosynthesis	1	1	map00140
Carbohydrate metabolism	Starch and sucrose metabolism	5	4	gmx00500
Carbohydrate metabolism	Galactose metabolism	3	2	gmx00052
Carbohydrate metabolism	Pentose and glucuronate interconversions	3	3	map00040
Carbohydrate metabolism	Amino sugar and nucleotide sugar metabolism	2	2	gmx00520
Carbohydrate metabolism	Ascorbate and aldarate metabolism	2	2	gmx00053
Energy metabolism	Nitrogen metabolism	1	1	gmx00910
Energy metabolism	Oxidative phosphorylation	1	1	map00190
Lipid metabolism	Linoleic acid metabolism	3	2	gmx00591
Lipid metabolism	alpha-Linolenic acid metabolism	2	1	gmx00592
Lipid metabolism	Fatty acid biosynthesis	2	2	map00061
Lipid metabolism	Arachidonic acid metabolism	1	1	gmx00590
Lipid metabolism	Glycerolipid metabolism	1	1	gmx00561
Metabolism of cofactors and vitamins	Thiamine metabolism	2	1	gmx00730
Metabolism of cofactors and vitamins	Retinol metabolism	1	1	map00830
Metabolism of cofactors and vitamins	Nicotinate and nicotin amide metabolism	1	1	map00760
Metabolism of other amino acids	Cyanoamino acid metabolism	1	1	map00460
Nucleotide metabolism	Purine metabolism	5	5	gmx00230
Nucleotide metabolism	Pyrimidine metabolism	2	2	gmx00240
Xenobiotics biodegradation and metabolism	Aminobenzoate degradation	1	1	map00627
Xenobiotics biodegradation and metabolism	Drug metabolism - cytochrome P450	1	1	map00982

Xenobiotics biodegradation and metabolism	Metabolism of xenobiotics by cytochrome P450	1	1	map00980

Supplementary Table 15 Over-represented KEGG pathways of the DEGs identified by the comparison between control and UV-B treatments in Cheongja 3

Pathway category	KEGG Pathway	number of sequence	Number of Enzyme	Pathway ID
Amino acid metabolism	Phenylalanine metabolism	6	7	gmx00360
Amino acid metabolism	Cysteine and methionine metabolism	4	5	gmx00270
Amino acid metabolism	Tryptophan metabolism	3	6	gmx00380
Amino acid metabolism	Phenylalanine, tyrosine and tryptophan biosynthesis	2	5	gmx00400
Amino acid metabolism	Alanine, aspartate and glutamate metabolism	1	1	gmx00250
Amino acid metabolism	Arginine and proline metabolism	1	1	gmx00330
Amino acid metabolism	Glycine, serine and threonine metabolism	1	1	gmx00260
Amino acid metabolism	Histidine metabolism	1	1	gmx00340
Amino acid metabolism	Lysine biosynthesis	1	2	gmx00300
Biosynthesis of other secondary metabolites	Phenylpropanoid biosynthesis	5	2	gmx00940
Biosynthesis of other secondary metabolites	Isoflavonoid biosynthesis	2	2	gmx00943
Biosynthesis of other secondary metabolites	Isoquinoline alkaloid biosynthesis	2	4	gmx00950
Biosynthesis of other secondary metabolites	Tropane, piperidine and pyridine alkaloid biosynthesis	2	5	gmx00960
Biosynthesis of other secondary metabolites	Anthocyanin biosynthesis	1	1	map00942
Biosynthesis of other secondary metabolites	Caffeine metabolism	1	1	gmx00232
Biosynthesis of other secondary metabolites	Flavonoid biosynthesis	1	1	gmx00941
Biosynthesis of other secondary metabolites	Novobiocin biosynthesis	1	4	map00760
Carbohydrate metabolism	Starch and sucrose metabolism	5	6	gmx00500
Carbohydrate metabolism	Pentose and glucuronate interconversions	4	3	gmx00040
Carbohydrate metabolism	Galactose metabolism	2	2	gmx00052
Carbohydrate metabolism	Amino sugar and nucleotide sugar metabolism	1	1	gmx00520
Carbohydrate metabolism	Ascorbate and aldarate metabolism	1	1	gmx00053

Carbohydrate metabolism	Glycolysis / Gluconeogenesis	1	1	gmx00010
Carbohydrate metabolism	Glyoxylate and dicarboxylate metabolism	1	1	gmx00630
Carbohydrate metabolism	Pyruvate metabolism	1	1	gmx00620
Energy metabolism	Carbon fixation in photosynthetic organisms	1	1	map00720
Energy metabolism	Sulfur metabolism	1	1	gmx00920
Energy metabolism	Oxidative phosphorylation	1	1	map00190
Lipid metabolism	Arachidonic acid metabolism	3	2	gmx00590
Lipid metabolism	Fatty acid biosynthesis	2	2	gmx00061
Lipid metabolism	Linoleic acid metabolism	2	2	gmx00591
Lipid metabolism	alpha-Linolenic acid metabolism	1	1	gmx00592
Lipid metabolism	Biosynthesis of unsaturated fatty acids	1	1	gmx01040
Lipid metabolism	Glycerolipid metabolism	1	1	gmx00561
Lipid metabolism	Steroid hormone biosynthesis	1	1	map00140
Lipid metabolism	Cutin, suberine and wax biosynthesis	1	1	map00073
Metabolism of cofactors and vitamins	Thiamine metabolism	4	1	gmx00730
Metabolism of cofactors and vitamins	Retinol metabolism	2	2	map00830
Metabolism of cofactors and vitamins	Biotin metabolism	1	1	gmx00780
Metabolism of cofactors and vitamins	Porphyrin and chlorophyll metabolism	1	1	gmx00620
Metabolism of cofactors and vitamins	Ubiquinone and other terpenoid-quinone biosynthesis	1	1	gmx00130
Metabolism of cofactors and vitamins	Nicotinate and nicotinamide metabolism	1	1	map00401
Metabolism of other amino acids	Glutathione metabolism	3	2	gmx00480
Metabolism of other amino acids	beta-Alanine metabolism	1	1	gmx00410
Metabolism of other amino acids	Cyanoamino acid metabolism	1	1	map00460
Metabolism of terpenoids and polyketides	Terpenoid backbone biosynthesis	1	1	gmx00900
Nucleotide metabolism	Purine metabolism	9	6	gmx00230
Nucleotide metabolism	Pyrimidine metabolism	2	2	gmx00240
Xenobiotics biodegradation and metabolism	Aminobenzoate degradation	1	1	map00627

Xenobiotics biodegradation and metabolism	Drug metabolism - cytochrome P450	1	1	map00982
Xenobiotics biodegradation and metabolism	Drug metabolism - other enzymes	1	1	map00983
Xenobiotics biodegradation and metabolism	Metabolism of xenobiotics by cytochrome P450	1	1	map00980

Supplementary Table 16 List of the DEGs from the Cheongja 3 vs Buseok comparison categorized by GO terms and their $\log_2$(fold change) values. ns: not significant, dash(-): uncalculated due to FPKM value = 0

Gene ID	Chromosome position	Gene definition	Cheongja 3 vs Buseok $\log_2$(fold change)		*A. thaliana* homolog
			0.5 h	6 h	
**Photosynthesis**					
Glyma09g07310	Gm09:6157087-6157613	chlorophyll A/B binding protein 1	ns	-1.39471	AT1G29930.1
Glyma09g08260	Gm09:7339729-7342548	photosystem I light harvesting complex gene 6	ns	-1.38303	AT1G19150.1
Glyma15g19810	Gm15:17240771-17243519	photosystem I light harvesting complex gene 6	ns	-1.14038	AT1G19150.1
Glyma16g27995	Gm16:31953083-31954592	light-harvesting chlorophyll-protein complex II subunit B1	ns	-1.92778	AT2G34430.1
Glyma11g35130	Gm11:36855886-36859844	light harvesting complex photosystem II	1.87061	ns	AT2G40100.1
Glyma04g33360	Gm04:38963142-38965295	light-harvesting chlorophyll-protein complex I subunit A4	1.48224	ns	AT3G47470.1
**Cell morphogenesis**					
Glyma13g26890	Gm13:30070997-30076596	Protein with RING/U-box and TRAF-like domains	ns	2.19223	AT3G61790.1
Glyma17g08020	Gm17:5928338-5930881	heat shock protein 70	ns	2.44538	AT3G12580.1
Glyma18g52480	Gm18:61075241-61082432	heat shock protein 70B	ns	1.14296	AT1G16030.1
Glyma02g10320	Gm02:8186067-8188789	heat shock protein 70	-6.05908	-4.15917	AT3G12580.1
Glyma08g02940	Gm08:2029877-2033833	Heat shock protein 70 (Hsp 70) family protein	-1.73003	ns	AT5G42020.1
Glyma15g09430	Gm15:6739539-6741346	heat shock cognate protein 70-1	-1.92982	ns	AT5G02500.1
Glyma05g36620	Gm05:40443106-40447303	heat shock protein 70	-1.59459	ns	AT3G12580.1
Glyma13g26890	Gm13:30070997-30076596	heat shock protein 70B	-3.25331	ns	AT1G16030.1
Glyma05g36600	Gm05:40426888-40430895	Heat shock protein 70 (Hsp 70) family protein	-1.55219	ns	AT5G42020.1
Glyma13g29591	Gm13:32478807-32481336	heat shock protein 70B	-1.99483	ns	AT1G16030.1
Glyma18g52471	Gm18:61067503-61071806	Protein with RING/U-box and TRAF-like domains	-1.17199	ns	AT3G61790.1
**Immune response**					
Glyma06g41880	Gm06:45152031-45155033	disease resistance protein (TIR-NBS-LRR class), putative	1.83743	ns	AT5G17680.1
Glyma16g33961	Gm16:36692020-36696860	disease resistance protein (TIR-NBS-LRR class), putative	2.05706	ns	AT5G17680.1

Glyma16g32321	Gm16:35526836-35530790	disease resistance protein (TIR-NBS-LRR class), putative	2.54783	ns	AT5G17680.1
Glyma12g16450	Gm12:15730001-15734533	disease resistance protein (TIR-NBS-LRR class), putative	1.28285	ns	AT5G17680.1
Glyma06g46665	Gm06:49244725-49251384	disease resistance protein (TIR-NBS-LRR class) family	ns	1.77505	AT5G36930.2
Glyma13g03770	Gm13:3846577-3852431	disease resistance protein (TIR-NBS-LRR class), putative	ns	2.81302	AT5G17680.1
Glyma16g25120	Gm16:29058174-29061521	disease resistance protein (TIR-NBS-LRR class), putative	2.21865	1.80936	AT5G17680.1
Glyma01g04000	Gm01:3487914-3494204	disease resistance protein (TIR-NBS-LRR class), putative	1.8517	1.5628	AT5G17680.1
Glyma03g07181	Gm03:7546779-7575160	disease resistance protein (TIR-NBS-LRR class) family	3.53008	3.95558	AT5G36930.2
Glyma16g34086	Gm16:36774650-36776469	disease resistance protein (TIR-NBS-LRR class), putative	2.08494	3.82768	AT5G17680.1
Glyma0220s50	scaffold_220:14-5602	disease resistance protein (TIR-NBS-LRR class) family	3.09462	2.29188	AT5G36930.2
Glyma16g00861	Gm16:516813-521800	disease resistance protein (TIR-NBS-LRR class) family	-1.57461	ns	AT5G41540.1
Glyma16g25071	Gm16:28995807-29006764	disease resistance protein (TIR-NBS-LRR class), putative	-5.92731	ns	AT5G17680.1
Glyma06g40950	Gm06:44230032-44239212	disease resistance protein (TIR-NBS-LRR class), putative	-1.76131	ns	AT5G17680.1
Glyma01g03921	Gm01:3390102-3396781	disease resistance protein (TIR-NBS-LRR class), putative	-3.62116	ns	AT5G17680.1
Glyma03g06285	Gm03:6480791-6481760	disease resistance protein (TIR-NBS-LRR class) family	-	-5.18062	AT5G44510.1
Glyma06g41404	Gm06:44686411-44690459	disease resistance protein (TIR-NBS-LRR class) family	-3.30704	-3.64384	AT5G45220.1
Glyma06g41700	Gm06:44984922-44988575	disease resistance protein (TIR-NBS-LRR class), putative	-7.85346	-7.9434	AT5G17680.1
Glyma16g33991	Gm16:36712977-36715288	disease resistance protein (TIR-NBS-LRR class) family	-3.78434	-3.00134	AT5G36930.2
Glyma07g07393	Gm07:6067981-6072205	disease resistance protein (TIR-NBS-LRR class), putative	-1.33937	-1.51589	AT5G17680.1
**Small molecule biosynthetic process**					
Glyma03g42140	Gm03:47422430-47424595	3-ketoacyl-CoA synthase 1	-1.09797	-1.67289	AT1G01120.1
Glyma12g09050	Gm12:6856671-6857958	arogenate dehydratase 6	ns	2.25158	AT1G08250.1
Glyma02g31971	Gm02:35217695-35218009	ATP synthase epsilon chain	1.09425	ns	ATCG00470.1
Glyma12g36098	Gm12:39209100-39209484	ATPase, F0 complex, subunit B/B\', bacterial/chloroplast	1.93141	ns	ATCG00130.1
Glyma15g11490	Gm15:8443116-8444805	ATPase, F1 complex, gamma subunit protein	-1.67689	-2.43976	AT4G04640.1
Glyma01g43880	Gm01:54742695-54744656	Chalcone and stilbene synthase family protein	-2.39674	ns	AT5G13930.1
Glyma11g01350	Gm11:793458-795686	Chalcone and stilbene synthase family protein	-1.47288	ns	AT5G13930.1
Glyma08g11610	Gm08:8462028-8464321	Chalcone and stilbene synthase family protein	-1.61131	1.26552	AT5G13930.1

Glyma08g11520	Gm08:8380331-8382218	Chalcone and stilbene synthase family protein	-2.16507	ns	AT5G13930.1
Glyma08g11635	Gm08:8475905-8477518	Chalcone and stilbene synthase family protein	-2.11658	ns	AT5G13930.1
Glyma05g28610	Gm05:34416316-34422554	Chalcone and stilbene synthase family protein	-1.65324	ns	AT5G13930.1
Glyma08g11530	Gm08:8384161-8386020	Chalcone and stilbene synthase family protein	ns	1.70509	AT5G13930.1
Glyma08g11620	Gm08:8466645-8469994	Chalcone and stilbene synthase family protein	ns	1.50531	AT5G13930.1
Glyma20g38560	Gm20:46127060-46129533	Chalcone-flavanone isomerase family protein	-1.55137	ns	AT3G55120.1
Glyma10g43850	Gm10:50409655-50413023	Chalcone-flavanone isomerase family protein	-2.39213	ns	AT3G55120.1
Glyma20g38580	Gm20:46134756-46136204	Chalcone-flavanone isomerase family protein	ns	1.33635	AT3G55120.1
Glyma19g03290	Gm19:3321966-3326662	chorismate mutase 1	-1.17906	ns	AT3G29200.1
Glyma04g06450	Gm04:4925679-4928196	chorismate mutase 2	ns	-2.73511	AT5G10870.1
Glyma07g00540	Gm07:258642-261286	D-3-phosphoglycerate dehydrogenase	-1.58202	ns	AT1G17745.1
Glyma10g40750	Gm10:48054737-48058055	D-3-phosphoglycerate dehydrogenase	-1.16952	ns	AT4G34200.1
Glyma15g14000	Gm15:10568140-10574343	fatty acid hydroxylase 1	3.26305	ns	AT2G34770.1
Glyma19g31120	Gm19:38858769-38898403	glutamate synthase 1	-1.28734	-1.51438	AT5G04140.1
Glyma02g39320	Gm02:44573639-44579555	glutamine-dependent asparagine synthase 1	2.12963	6.11727	AT3G47340.1
Glyma14g37440	Gm14:46713760-46720531	glutamine-dependent asparagine synthase 1	ns	2.9884	AT3G47340.1
Glyma02g39320	Gm02:44573639-44579555	glutamine-dependent asparagine synthase 1	ns	3.55396	AT3G47340.1
Glyma15g06120	Gm15:4337691-4343365	GTP cyclohydrolase II	-1.51303	ns	AT5G64300.1
Glyma13g08035	Gm13:8281822-8290126	GTP cyclohydrolase II	ns	-1.23002	AT5G64300.1
Glyma05g01460	Gm05:960778-967307	H(+)-ATPase 11	ns	-1.29312	AT5G62670.1
Glyma14g06780	Gm14:5001487-5006600	L-Aspartase-like family protein	-1.55519	ns	AT1G36280.1
Glyma19g05570	Gm19:6087841-6091946	Methylenetetrahydrofolate reductase family protein	1.26601	1.84297	AT5G38710.1
Glyma18g51400	Gm18:60300025-60303451	Methylenetetrahydrofolate reductase family protein	ns	2.85482	AT5G38710.1
Glyma08g14670	Gm08:10680461-10685058	myo-inositol-1-phosphate synthase 1	1.86715	ns	AT4G39800.1
Glyma18g02210	Gm18:1364734-1367733	myo-inositol-1-phosphate synthase 2	ns	2.13603	AT2G22240.1
Glyma08g14670	Gm08:10680461-10685058	myo-inositol-1-phosphate synthase 3	1.45111	1.50415	AT5G10170.1
Glyma18g02210	Gm18:1364734-1367733	myo-inositol-1-phosphate synthase 3	ns	3.92076	AT5G10170.1

Glyma05g31450	Gm05:36578951-36583516	myo-inositol-1-phosphate synthase 3	ns	3.77442	AT5G10170.1
Glyma05g31450	Gm05:36578951-36583516	myo-inositol-1-phosphate synthase 3	ns	-4.09143	AT5G10170.1
Glyma05g03010.1	Gm05:2295964-2300224	nucleoside diphosphate kinase 2	ns	-1.20196	AT5G63310.1
Glyma03g28476	Gm03:36406523-36411505	pyrroline-5- carboxylate (P5C) reductase	ns	-2.4525	AT5G14800.1
Glyma12g04480	Gm12:2981850-2987782	thymidylate synthase 1	-1.96516	ns	AT2G16370.1
**Fatty acid biosynthetic process**					
Glyma15g38490	Gm15:44920076-44924919	MAP kinase 19	ns	1.30341	AT3G14720.1
Glyma14g03190	Gm14:2035901-2041951	MAP kinase 20	ns	1.33972	AT2G42880.1
Glyma11g15700	Gm11:11369715-11373376	mitogen-activated protein kinase 3	-1.47132	ns	AT3G45640.1
Glyma11g02950	Gm11:1927110-1933660	hydroxymethylglutaryl-CoA synthase	ns	1.4018	AT4G11820.2
Glyma18g12660	Gm18:11874766-11879530	rhamnose biosynthesis 1	1.44473	1.24404	AT1G78570.1
Glyma18g12660	Gm18:11874766-11879530	rhamnose biosynthesis 1	2.01043	2.2498	AT1G78570.1
Glyma05g28510	Gm05:34318927-34320836	UDP-D-glucuronate 4-epimerase 6	1.30222	ns	AT3G23820.1
Glyma18g06510	Gm18:5074644-5079669	NAD(P)-binding Rossmann-fold superfamily protein	1.46454	ns	AT5G58490.1
Glyma11g29460	Gm11:30155459-30160850	NAD(P)-binding Rossmann-fold superfamily protein	2.94344	ns	AT5G58490.1
Glyma09g40580	Gm09:45474399-45477120	NAD(P)-binding Rossmann-fold superfamily protein	-2.84626	-1.93939	AT2G45400.1
Glyma18g45250	Gm18:55008998-55011687	NAD(P)-binding Rossmann-fold superfamily protein	-6.2116	-5.09622	AT2G45400.1
Glyma09g40590	Gm09:45480404-45483317	NAD(P)-binding Rossmann-fold superfamily protein	-2.8206	ns	AT2G45400.1
Glyma12g02250	Gm12:1389392-1395672	NAD(P)-binding Rossmann-fold superfamily protein	-2.15826	ns	AT5G19440.1
Glyma08g23120	Gm08:17611698-17614488	NAD(P)-binding Rossmann-fold superfamily protein	-1.79769e+308	ns	AT2G33590.1
Glyma13g44700	Gm13:44081559-44084297	cinnamoyl coa reductase	-1.0674	ns	AT1G80820.1
**Signaling**					
Glyma17g08510	Gm17:6296131-6303862	diacylglycerol kinase1 (DGK 1)	ns	1.217	AT5G07920.1
Glyma08g20830	Gm08:15811469-15819531	Protein phosphatase 2A, regulatory subunit PR55 (ABI 1)	ns	3.03934	AT1G17720.1
Glyma19g24375	Gm19:29931574-29938227	Protein phosphatase 2A regulatory B subunit family protein (ABI1)	ns	2.3848	AT4G15415.1
Glyma18g03090	Gm18:2033237-2038137	phospholipase C 2 (PI-PLC 2)	0.038	-1.558	AT3G08510.1
Glyma03g38620	Gm03:44925729-44930360	phytochrome A	2.43949	ns	AT1G09570.1

Glyma04g40640	Gm04:46630389-46635618	pseudo-response regulator 5	ns	1.35497	AT5G24470.1
Glyma05g05260	Gm05:4565282-4568952	RAB GTPase homolog 1C	1.39336	ns	AT4G17530.1
Glyma05g08260	Gm05:8219985-8222377	RAS-related nuclear protein-1	1.73081	ns	AT5G20010.1
Glyma18g49540	Gm18:58932313-58933700	Calcium-binding EF-hand family protein	-2.46944	ns	AT5G39670.1
Glyma19g43370	Gm19:49038174-49039551	calmodulin-like 11	-3.46234	ns	AT3G22930.1
Glyma19g44970	Gm19:50244045-50249070	pseudo-response regulator 9	1.52628	1.55819	AT2G46790.1
**Oxidation reduction**					
Glyma09g26661	Gm09:33218707-33223656	cytochrome P450, family 716, subfamily A, polypeptide 2	3.31325	-	AT5G36140.1
Glyma05g02730	Gm05:2090766-2093137	cytochrome P450, family 71, subfamily A, polypeptide 20	1.72076	2.43777	AT4G13310.1
Glyma03g03560	Gm03:3344404-3346855	cytochrome P450, family 83, subfamily B, polypeptide 1	1.56415	2.15742	AT4G31500.1
Glyma03g18430	Gm03:23312388-23315708	NAD(P)-linked oxidoreductase superfamily protein	1.55348	1.49178	AT2G37770.2
Glyma18g40690	Gm18:49384186-49404912	NAD(P)-linked oxidoreductase superfamily protein	1.47257	1.30663	AT2G37770.2
Glyma14g39790	Gm14:48884565-48887025	12-oxophytodienoate reductase 1	1.40065	1.13983	AT1G76680.1
Glyma03g03630	Gm03:3430118-3432394	cytochrome P450, family 83, subfamily B, polypeptide 1	1.27766	1.39273	AT4G31500.1
Glyma19g05570	Gm19:6087841-6091946	Methylenetetrahydrofolate reductase family protein	1.26601	1.84297	AT5G38710.1
Glyma15g14000	Gm15:10568140-10574343	fatty acid hydroxylase 1	3.26305	ns	AT2G34770.1
Glyma03g34821	Gm03:42125961-42126684	Thioredoxin superfamily protein	2.43344	ns	AT3G52960.1
Glyma13g16950	Gm13:20811386-20813635	oxophytodienoate-reductase 3	1.91094	ns	AT2G06050.1
Glyma14g33490	Gm14:41458475-41463776	nitrate reductase 1	1.86479	ns	AT1G77760.1
Glyma15g02230	Gm15:1488816-1494705	NADP-malic enzyme 4	1.76734	ns	AT1G79750.1
Glyma06g01850	Gm06:1169726-1172962	glyceraldehyde-3-phosphate dehydrogenase B subunit	1.40143	ns	AT1G42970.1
Glyma05g28510	Gm05:34318927-34320836	UDP-D-glucuronate 4-epimerase 6	1.30222	ns	AT3G23820.1
Glyma06g34940	Gm06:36668157-36673471	homogentisate 1,2-dioxygenase	1.28568	ns	AT5G54080.1
Glyma18g51260	Gm18:60197966-60201157	6-phosphogluconate dehydrogenase family protein	-1.33899	ns	AT3G02360.1
Glyma04g03780	Gm04:2762653-2764820	cytochrome P450, family 82, subfamily C, polypeptide 4	-1.53994	ns	AT4G31940.1
Glyma07g00540	Gm07:258642-261286	D-3-phosphoglycerate dehydrogenase	-1.58202	ns	AT1G17745.1
Glyma10g38940	Gm10:46671848-46675291	RmlC-like cupins superfamily protein	-1.63264	ns	AT4G14710.1

Glyma06g40790	Gm06:43976715-43980827	NAD(P)-linked oxidoreductase superfamily protein	-1.95134	ns	AT1G60710.1
Glyma11g19190	Gm11:15880582-15889215	Jojoba acyl CoA reductase-related male sterility protein	-1.9541	ns	AT4G33790.1
Glyma15g02790	Gm15:1939035-1946519	Glucose-methanol-choline (GMC) oxidoreductase family protein	-2.02076	ns	AT1G72970.1
Glyma06g21920	Gm06:18534605-18541507	Cytochrome P450 superfamily protein	-2.07295	ns	AT5G07990.1
Glyma19g32650	Gm19:40386812-40389246	cytochrome P450, family 93, subfamily D, polypeptide 1	-2.0909	ns	AT5G06900.1
Glyma14g01880	Gm14:1083679-1086440	cytochrome p450, family 71, subfamily B, polypeptide 11	-3.61226	ns	AT5G25120.1
Glyma16g32010	Gm16:35215938-35218572	cytochrome P450, family 71, subfamily A, polypeptide 26	-4.05028	-3.37396	AT3G48270.1
Glyma02g05470	Gm02:4397256-4400056	flavanone 3-hydroxylase	-3.27211	-2.08189	AT3G51240.1
Glyma10g34460	Gm10:42610680-42615095	cytochrome P450, family 76, subfamily C, polypeptide 4	-2.56216	-1.89641	AT2G45550.1
Glyma11g13070	Gm11:9325682-9327781	allene oxide synthase	-1.07797	-1.25998	AT5G42650.1
Glyma06g12780	Gm06:9979014-9982400	GroES-like zinc-binding dehydrogenase family protein	ns	3.39962	AT5G43940.1
Glyma20g00980	Gm20:678854-681696	cytochrome P450, family 71, subfamily B, polypeptide 34	ns	3.01834	AT3G26300.1
Glyma18g51400	Gm18:60300025-60303451	Methylenetetrahydrofolate reductase family protein	ns	2.85482	AT5G38710.1
Glyma13g28320	Gm13:31364576-31366099	methionine sulfoxide reductase B 2	ns	2.60089	AT4G21860.1
Glyma20g24810	Gm20:34452631-34454918	cinnamate-4-hydroxylase	ns	2.17036	AT2G30490.1
Glyma04g36373	Gm04:42913721-42915643	cytochrome P450, family 76, subfamily C, polypeptide 2	ns	2.16315	AT2G45570.1
Glyma15g05580	Gm15:3950825-3953292	cytochrome P450, family 71, subfamily B, polypeptide 37	ns	2.02217	AT3G26330.1
Glyma02g05450	Gm02:4388741-4392353	flavanone 3-hydroxylase	ns	1.70143	AT3G51240.1
Glyma19g01160	Gm19:811042-813940	Oxidoreductase, zinc-binding dehydrogenase family protein	ns	1.64174	AT1G23740.1
Glyma19g32880	Gm19:40562660-40565087	cytochrome P450, family 93, subfamily D, polypeptide 1	ns	1.53566	AT5G06900.1
Glyma03g40280	Gm03:46076954-46080391	copper/zinc superoxide dismutase 1	ns	1.48004	AT1G08830.1
Glyma19g42890	Gm19:48706602-48710141	copper/zinc superoxide dismutase 1	ns	1.43929	AT1G08830.1
Glyma13g33620	Gm13:35411937-35415140	cytochrome P450, family 72, subfamily A, polypeptide 7	ns	1.43626	AT3G14610.1
Glyma17g14320	Gm17:11077645-11082163	cytochrome P450, family 706, subfamily A, polypeptide 4	ns	1.37776	AT4G12300.1
Glyma07g05820	Gm07:4508512-4510870	cytochrome P450, family 78, subfamily A, polypeptide 6	ns	1.37723	AT2G46660.1
Glyma14g38580	Gm14:47721241-47725350	cinnamate-4-hydroxylase	ns	1.23804	AT2G30490.1
Glyma07g32330	Gm07:37261035-37263430	cytochrome P450, family 93, subfamily D, polypeptide 1	ns	1.14103	AT5G06900.1

Glyma06g18551	Gm06:14848334-14851495	cytochrome P450, family 71, subfamily A, polypeptide 22	ns	-1.1561	AT3G48310.1
Glyma17g09860	Gm17:7359682-7364912	aldehyde dehydrogenase 2B4	ns	-1.16649	AT3G48000.1
Glyma12g36780	Gm12:39823419-39827808	cytochrome P450, family 712, subfamily A, polypeptide 1	ns	-1.18379	AT2G42250.1
Glyma14g10890	Gm14:9085876-9088071	Fatty acid/sphingolipid desaturase	ns	-1.23884	AT2G46210.1
Glyma05g03580	Gm05:2763458-2772019	Glucose-methanol-choline (GMC) oxidoreductase family protein	ns	-1.28927	AT1G12570.1
Glyma02g37160	Gm02:42491529-42497799	ascorbate peroxidase 4	ns	-1.35074	AT4G09010.1
Glyma09g31820	Gm09:38432576-38435991	Cytochrome P450 superfamily protein	ns	-1.40214	AT5G07990.1
Glyma15g13090	Gm15:9750359-9757901	ferric reduction oxidase 7	ns	-1.48904	AT5G49740.1
Glyma02g09630	Gm02:7570010-7574618	Fe superoxide dismutase 2	ns	-1.93341	AT5G51100.1
Glyma03g28476	Gm03:36406523-36411505	pyrroline-5- carboxylate (P5C) reductase	ns	-2.4525	AT5G14800.1
Glyma14g04530	Gm14:3114028-3120767	Plant L-ascorbate oxidase	ns	-2.56758	AT5G21105.1
Glyma16g08460	Gm16:7860157-7865392	NADP-malic enzyme 4	ns	-4.40365	AT1G79750.1

Supplementary Table 17 List of the DEGs from the comparison of control vs 0.5 & 6 h UV-B treatment in Cheongja 3 categorized by GO terms and their log_2(fold change) values. ns: not significant, dash(-): uncalculated due to FPKM value=0

Gene ID	Chromosome position	Gene definition	FPKM value		Log_2 (fold change)	*A. thaliana* homolog
			0 h	0.5 h		
**Metal ion (C vs 0.5 h)**						
Glyma10g37500	Gm10:45475418-45476872	Heavy metal transport/detoxification superfamily protein	28.3029	265.753	3.231064189	AT1G01490.1
Glyma12g08440	Gm12:6185227-6187696	Heavy metal transport/detoxification superfamily protein	7.4979	92.5606	3.625839727	AT5G60800.1
Glyma09g24190	Gm09:29918018-29919111	Heavy metal transport/detoxification superfamily protein	7.95928	157.166	4.303507411	AT1G01490.1
Glyma16g29690	Gm16:33423740-33425662	Heavy metal transport/detoxification superfamily protein	0.595342	11.9561	4.327884382	AT1G01490.1
Glyma05g26330	Gm05:32310948-32316398	copper-exporting ATPase	3.42656	111.476	5.023828382	AT5G44790.1
Glyma16g29670	Gm16:33413332-33414359	Heavy metal transport/detoxification superfamily protein	2.7382	114.637	5.387701124	AT1G01490.1
Glyma11g20030	Gm11:16846077-16848107	Heavy metal transport/detoxification superfamily protein	12.1597	540.354	5.473725324	AT5G60800.2
Glyma16g29710	Gm16:33432554-33433447	Heavy metal transport/detoxification superfamily protein	6.74862	358.703	5.73205348	AT1G01490.1
Glyma16g29701	Gm16:33428772-33429954	Heavy metal transport/detoxification superfamily protein	3.33598	202.356	5.922641177	AT1G01490.1
**Oxidation reduction (C vs 6 h)**						
Glyma14g14520	Gm14:14829121-14832116	cytochrome P450, family 71 subfamily B, polypeptide 7	19.7329	0.606759	-5.023335581	AT1G13110.1
Glyma06g12340	Gm06:9526563-9528716	2-oxoglutarate (2OG) and Fe(II)-dependent oxygenase superfamily protein	204.121	8.59855	-4.56918741	AT1G77330.1
Glyma17g03030	Gm17:2026509-2029231	NAD(P)-binding Rossmann-fold superfamily protein	26.1027	1.11947	-4.543311271	AT3G62830.1
Glyma16g29790	Gm16:33472524-33475361	beta-hydroxylase 1	52.6946	3.89929	-3.756371765	AT4G25700.1
Glyma05g00510	Gm05:242320-244872	Cytochrome P450 superfamily protein	19.3587	1.59266	-3.603471855	AT5G07990.1
Glyma03g03560	Gm03:3344404-3346855	cytochrome P450, family 83, subfamily B, polypeptide 1	23.6899	2.09266	-3.500862271	AT4G31500.1
Glyma09g02170	Gm09:1444943-1450815	ferric reduction oxidase 7	80.5393	7.26485	-3.470688018	AT5G49740.1
Glyma08g19170	Gm08:14466235-14468766	Peroxidase superfamily protein	53.8603	5.25881	-3.356413987	AT5G64120.1
Glyma13g04410	Gm13:4741878-4749355	Peroxidase superfamily protein	17.9547	1.93955	-3.210567675	AT3G01420.1
Glyma07g31380	Gm07:36400303-36405352	cytochrome P450, family 71, subfamily A, polypeptide 26	18.0273	2.13841	-3.075572942	AT3G48270.1

Glyma19g01210	Gm19:830857-833715	formate dehydrogenase	25.0986	3.25294	-2.947790775	AT5G14780.1
Glyma15g05580	Gm15:3950825-3953292	cytochrome P450, family 71, subfamily B, polypeptide 37	17.7466	2.64305	-2.747267028	AT3G26330.1
Glyma13g28670	Gm13:31663733-31665547	NAD(P)-binding Rossmann-fold superfamily protein	41.7248	6.34454	-2.717317661	AT5G18660.1
Glyma17g14320	Gm17:11077645-11082163	cytochrome P450, family 706, subfamily A, polypeptide 4	95.8637	14.7721	-2.69810969	AT4G12300.1
Glyma07g00240	Gm07:60819-70613	high chlorophyll fluorescence phenotype 173	28.5134	4.43102	-2.685929336	AT1G16720.1
Glyma16g08470	Gm16:7865858-7871116	2-oxoglutarate (2OG) and Fe(II)-dependent oxygenase superfamily protein	32.9671	5.91955	-2.477467571	AT1G35190.1
Glyma13g33620	Gm13:35411937-35415140	cytochrome P450, family 72, subfamily A, polypeptide 15	10.7322	90.4791	3.075638734	AT3G14690.1
Glyma15g24010	Gm15:24503356-24506971	flavin-dependent monooxygenase 1	4.03798	40.2882	3.31865168	AT1G19250.1
Glyma02g06400	Gm02:5082891-5090808	succinate dehydrogenase 1-1	9.26411	96.4453	3.379986645	AT5G66760.1
Glyma09g41440	Gm09:46112212-46114868	Peroxidase superfamily protein	3.39595	42.3893	3.64181302	AT5G05340.1
Glyma11g06690	Gm11:4727142-4729773	cytochrome P450, family 71, subfamily B, polypeptide 34	4.01362	55.189	3.781404807	AT3G26300.1
Glyma17g01580	Gm17:956566-961506	SKU5 similar 4	2.10524	30.6921	3.865810744	AT4G22010.1
Glyma01g42840	Gm01:53990059-53991492	glutathione peroxidase 6	8.17628	123.847	3.920970509	AT4G11600.1
Glyma11g02630	Gm11:1719472-1720899	glutathione peroxidase 6	4.22072	67.3568	3.99626257	AT4G11600.1
Glyma11g00550	Gm11:197381-205108	gibberellin 2-oxidase 8	2.98576	56.1372	4.232786998	AT4G21200.1
Glyma03g24020	Gm03:30681540-30684519	alkenal reductase	8.72661	166.224	4.251563565	AT5G16970.1

Supplementary Table 18 List of the DEGs from the comparison of control vs 0.5 h UV-B treatment in Buseok categorized by GO terms and their log_2(fold change) values. ns: not significant, dash(-): uncalculated due to FPKM value = 0

Gene ID	Chromosome position	Gene definition	FPKM value		Log_2 (fold change)	*A. thaliana* homolog
			0 h	0.5 h		
**Apoptosis**						
Glyma03g14888	Gm03:19079981-19089860	Disease resistance protein (TIR-NBS-LRR class) family	2.12669	38.1191	4.1638324	AT5G36930.2
Glyma06g40690	Gm06:43857935-43861836	disease resistance protein (TIR-NBS-LRR class), putative	1.11115	14.2975	3.685637414	AT5G17680.1
Glyma06g41880	Gm06:45152031-45155033	disease resistance protein (TIR-NBS-LRR class), putative	2.13785	25.9722	3.602735688	AT5G17680.1
Glyma06g40740	Gm06:43913599-43918380	Disease resistance protein (TIR-NBS-LRR class) family	2.44038	69.3254	4.828206318	AT4G12010.1
Glyma16g33590	Gm16:36465825-36471050	Disease resistance protein (TIR-NBS-LRR class) family	4.84064	66.4426	3.77883882	AT5G36930.2
Glyma05g17460	Gm05:20185056-20190951	Disease resistance protein (CC-NBS-LRR class) family	1.72206	21.3444	3.631650293	AT5G66900.1
Glyma12g16450	Gm12:15730001-15734533	disease resistance protein (TIR-NBS-LRR class), putative	1.70778	16.3815	3.261873425	AT5G17680.1
Glyma17g21240	Gm17:20538055-20543536	Disease resistance protein (CC-NBS-LRR class) family	2.75143	30.1344	3.45315781	AT5G66900.1
Glyma16g25120	Gm16:29058174-29061521	disease resistance protein (TIR-NBS-LRR class), putative	4.89443	71.7237	3.873237154	AT5G17680.1
Glyma18g09220	Gm18:8096589-8100424	NB-ARC domain-containing disease resistance protein	0.373772	5.99216	4.002845743	AT3G07040.1
Glyma18g51741	Gm18:60526240-60534001	Cysteine proteinases superfamily protein	0.379668	8.94621	4.558466311	AT1G09730.1
**Signaling**						
Glyma16g25120	Gm16:29058174-29061521	disease resistance protein (TIR-NBS-LRR class), putative	4.89443	71.7237	3.873237154	AT5G17680.1
Glyma12g16450	Gm12:15730001-15734533	disease resistance protein (TIR-NBS-LRR class), putative	1.70778	16.3815	3.261873425	AT5G17680.1
Glyma06g39760	Gm06:42662480-42670185	diacylglycerol kinase 5	0.595198	15.3667	4.690293891	AT2G20900.2
Glyma06g40690	Gm06:43857935-43861836	disease resistance protein (TIR-NBS-LRR class), putative	1.11115	14.2975	3.685637414	AT5G17680.1
Glyma02g41300	Gm02:46466665-46468249	Calcium-binding EF-hand family protein	1.85606	37.807	4.348338122	AT1G18210.1
Glyma18g44450	Gm18:54146609-54150302	SOS3-interacting protein 1	35.6031	5.45397	-2.706624194	AT5G58380.1
Glyma06g41880	Gm06:45152031-45155033	disease resistance protein (TIR-NBS-LRR class), putative	2.13785	25.9722	3.602735688	AT5G17680.1
Glyma06g40740	Gm06:43913599-43918380	disease resistance protein (TIR-NBS-LRR class) family	2.44038	69.3254	4.828206318	AT4G12010.1
Glyma14g39660	Gm14:48710869-48712618	Calcium-binding EF-hand family protein	1.46972	22.7162	3.950108284	AT1G18210.1

Glyma16g33590	Gm16:36465825-36471050	disease resistance protein (TIR-NBS-LRR class) family	4.84064	66.4426	3.77883882	AT5G36930.2
Glyma03g14888	Gm03:19079981-19089860	disease resistance protein (TIR-NBS-LRR class) family	2.12669	38.1191	4.1638324	AT5G36930.2
Glyma08g45920	Gm08:45173313-45177284	RAB GTPase homolog A5B	2.24043	24.4431	3.44757971	AT3G07410.1
**Biological regulation**						
Glyma17g35490	Gm17:39478720-39481808	Subtilase family protein	16.2025	1.05944	-3.934842644	AT2G20900.2
Glyma14g09670	Gm14:7731750-7734701	Subtilase family protein	16.5742	1.62386	-3.351440074	AT4G14550.1
Glyma05g04920	Gm05:4127453-4128819	Integrase-type DNA-binding superfamily protein	23.3206	2.39367	-3.284308731	AT4G27410.2
Glyma06g01430	Gm06:888837-891458	cryptochrome-interacting basic-helix-loop-helix 1	6.41691	0.740769	-3.114783118	AT2G17040.1
Glyma19g28550	Gm19:36085223-36087778	atypical CYS HIS rich thioredoxin 4	63.8687	7.68678	-3.054657802	AT5G17680.1
Glyma20g29250	Gm20:38156769-38163260	K-box region and MADS-box transcription factor family protein	61.5593	8.99979	-2.774013583	AT5G67360.1
Glyma19g34380	Gm19:41985092-41988819	indole-3-acetic acid inducible 14	48.7968	7.19256	-2.762209286	AT1G18210.1
Glyma18g44450	Gm18:54146609-54150302	SOS3-interacting protein 1	35.6031	5.45397	-2.706624194	AT5G58380.1
Glyma03g31530	Gm03:39420173-39423838	indole-3-acetic acid inducible 14	55.8951	9.24764	-2.595564674	AT3G58120.1
Glyma05g20710	Gm05:24873226-24875484	WRKY DNA-binding protein 15	11.5381	89.334	2.952803689	AT1G32640.1
Glyma01g12740	Gm01:15937028-15939729	Basic helix-loop-helix (bHLH) DNA-binding family protein	3.51902	27.7873	2.981180041	AT4G11070.1
Glyma08g02580	Gm08:1759379-1761625	WRKY family transcription factor	4.05402	33.4848	3.04608124	AT5G17680.1
Glyma15g08520	Gm15:6016523-6018064	Thioredoxin superfamily protein	4.0496	33.7285	3.058116841	AT4G12010.1
Glyma16g02960	Gm16:2538809-2542188	WRKY family transcription factor	8.48946	71.7288	3.078807801	AT1G18210.1
Glyma02g40320	Gm02:45555892-45557816	cytokinin response factor 4	3.08423	27.201	3.14067743	AT5G36930.2
Glyma07g06320	Gm07:5052596-5055648	WRKY family transcription factor	5.78447	53.8381	3.218370813	AT2G44940.1
Glyma12g16450	Gm12:15730001-15734533	disease resistance protein (TIR-NBS-LRR class), putative	1.70778	16.3815	3.261873425	AT2G38470.1
Glyma07g14750	Gm07:14558213-14560706	GATA transcription factor 2	2.26097	23.9317	3.403909128	AT4G14550.1
Glyma08g45920	Gm08:45173313-45177284	RAB GTPase homolog A5B	2.24043	24.4431	3.44757971	AT4G27950.1
Glyma17g23740	Gm17:23912235-23914953	NAC domain containing protein 83	8.61517	96.2363	3.481630006	AT3G15210.1
Glyma04g38560	Gm04:44929347-44935783	NAC (No Apical Meristem) domain transcriptional regulator superfamily protein	3.79691	43.2005	3.508150303	AT2G22300.1
Glyma12g35550	Gm12:38675212-38676429	ethylene responsive element binding factor 4	8.20849	98.4863	3.584734292	AT1G80840.1
Glyma06g41880	Gm06:45152031-45155033	disease resistance protein (TIR-NBS-LRR class), putative	2.13785	25.9722	3.602735688	AT5G17680.1

Glyma16g01940	Gm16:1443745-1447223	NAC transcription factor-like 9	3.98276	48.8983	3.617943858	AT2G45050.1
Glyma06g40690	Gm06:43857935-43861836	disease resistance protein (TIR-NBS-LRR class), putative	1.11115	14.2975	3.685637414	AT4G36990.1
Glyma06g38410	Gm06:41306756-41309130	NAC (No Apical Meristem) domain transcriptional regulator superfamily protein	1.53635	20.651	3.748632821	AT1G01720.1
Glyma16g33590	Gm16:36465825-36471050	disease resistance protein (TIR-NBS-LRR class) family	4.84064	66.4426	3.77883882	AT4G17500.1
Glyma05g05180	Gm05:4460843-4462512	ethylene responsive element binding factor 1	2.15133	30.9907	3.848534595	AT5G17680.1
Glyma13g35550	Gm13:36983073-36984757	NAC domain containing protein 3	4.562	65.7679	3.849645253	AT5G13180.1
Glyma16g25120	Gm16:29058174-29061521	disease resistance protein (TIR-NBS-LRR class), putative	4.89443	71.7237	3.873237154	AT2G23320.1
Glyma05g36970	Gm05:40700821-40702765	WRKY family transcription factor	5.12028	76.4193	3.899642432	AT4G23810.1
Glyma08g16190	Gm08:11809098-11812372	basic helix-loop-helix (bHLH) DNA-binding superfamily protein	2.52337	38.1929	3.9198808	AT2G42280.1
Glyma14g39660	Gm14:48710869-48712618	Calcium-binding EF-hand family protein	1.46972	22.7162	3.950108284	AT4G35580.2
Glyma11g10230	Gm11:7351786-7354368	NAC domain containing protein 36	1.86014	28.9262	3.958893698	AT1G28480.1
Glyma12g14130	Gm12:12824893-12828853	Basic-leucine zipper (bZIP) transcription factor family protein	1.02201	16.2708	3.992803969	AT5G36930.2
Glyma06g16440	Gm06:12910300-12912252	NAC (No Apical Meristem) domain transcriptional regulator superfamily protein	4.08426	65.0257	3.99286341	AT4G17500.1
Glyma03g14888	Gm03:19079981-19089860	disease resistance protein (TIR-NBS-LRR class) family	2.12669	38.1191	4.1638324	AT4G34530.1
Glyma02g41300	Gm02:46466665-46468249	Calcium-binding EF-hand family protein	1.85606	37.807	4.348338122	AT1G08570.1
Glyma02g01960	Gm02:1429083-1430853	Integrase-type DNA-binding superfamily protein	2.43653	52.061	4.417303133	AT5G67360.1
Glyma17g15480	Gm17:12209107-12210493	ethylene responsive element binding factor 1	1.52409	34.6366	4.506277315	AT4G23810.1
Glyma15g00570	Gm15:289708-291897	WRKY DNA-binding protein 40	4.74617	108.889	4.519950633	AT4G23810.1
Glyma01g39260	Gm01:51227462-51229777	heat shock factor 4	1.05557	24.5138	4.537499981	AT2G47520.1
Glyma04g40450	Gm04:46495020-46498562	NAC transcription factor-like 9	6.18144	157.695	4.673050146	AT1G24260.1
Glyma06g39760	Gm06:42662480-42670185	diacylglycerol kinase 5	0.595198	15.3667	4.690293891	AT3G15500.1
Glyma01g31921	Gm01:43157131-43160950	WRKY DNA-binding protein 33	0.766895	20.0552	4.70880348	AT3G07410.1
Glyma06g40740	Gm06:43913599-43918380	disease resistance protein (TIR-NBS-LRR class) family	2.44038	69.3254	4.828206318	AT4G35580.2
Glyma05g31190	Gm05:36357224-36365346	signal responsive 1	0.839952	32.7855	5.286607201	AT1G01720.1
